# Rapid Identification of New Biomarkers for the Classification of GM1 Type 2 Gangliosidosis Using an Unbiased ^1^H NMR-Linked Metabolomics Strategy

**DOI:** 10.3390/cells10030572

**Published:** 2021-03-05

**Authors:** Benita C. Percival, Yvonne L. Latour, Cynthia J. Tifft, Martin Grootveld

**Affiliations:** 1 Leicester School of Pharmacy, De Montfort University, The Gateway, Leicester LE1 9BH, UK; P11279990@alumni365.dmu.ac.uk; 2 Department of Pathology, Microbiology, and Immunology, Vanderbilt University, Nashville, TN 37232-0252, USA; Yvonne.l.latour@vanderbilt.edu; 3 Deputy Clinical Director, National Human Genome Research Institute, Director, National Institutes of Health, Bethesda, MD 20892-1205, USA; cynthiat@mail.nih.gov

**Keywords:** GM1 gangliosidosis, lysosomal storage disorders, nuclear magnetic resonance (NMR) analysis, NMR-based metabolomics, biomarkers, validation, metabolite set enrichment analysis

## Abstract

Biomarkers currently available for the diagnosis, prognosis, and therapeutic monitoring of GM1 gangliosidosis type 2 (GM1T2) disease are mainly limited to those discovered in targeted proteomic-based studies. In order to identify and establish new, predominantly low-molecular-mass biomarkers for this disorder, we employed an untargeted, multi-analyte approach involving high-resolution ^1^H NMR analysis coupled to a range of multivariate analysis and computational intelligence technique (CIT) strategies to explore biomolecular distinctions between blood plasma samples collected from GM1T2 and healthy control (HC) participants (*n* = 10 and 28, respectively). The relationship of these differences to metabolic mechanisms underlying the pathogenesis of GM1T2 disorder was also investigated. ^1^H NMR-linked metabolomics analyses revealed significant GM1T2-mediated dysregulations in ≥13 blood plasma metabolites (corrected *p* < 0.04), and these included significant upregulations in 7 amino acids, and downregulations in lipoprotein-associated triacylglycerols and alanine. Indeed, results acquired demonstrated a profound distinctiveness between the GM1T2 and HC profiles. Additionally, employment of a genome-scale network model of human metabolism provided evidence that perturbations to propanoate, ethanol, amino-sugar, aspartate, seleno-amino acid, glutathione and alanine metabolism, fatty acid biosynthesis, and most especially branched-chain amino acid degradation (*p* = 10^−12^−10^−5^) were the most important topologically-highlighted dysregulated pathways contributing towards GM1T2 disease pathology. Quantitative metabolite set enrichment analysis revealed that pathological locations associated with these dysfunctions were in the order fibroblasts > Golgi apparatus > mitochondria > spleen ≈ skeletal muscle ≈ muscle in general. In conclusion, results acquired demonstrated marked metabolic imbalances and alterations to energy demand, which are consistent with GM1T2 disease pathogenesis mechanisms.

## 1. Introduction

Gangliosidoses represent lysosomal storage disorders (LSDs) arising from the adverse accumulation of GM1 or GM2 gangliosides; GM1 gangliosidosis (MIM# 230500) has both central nervous system (CNS) and systemic findings, whilst GM2 disorders (including Tay-Sachs and Sandhoff diseases) are essentially limited to the former. Both diseases have autosomal recessive inheritance modes, and are characterized by a series of sequential clinical presentations, which range from a severe infantile form to a milder, chronic adult one. Both GM1 and GM2 disorders are debilitating diseases which are currently still without any available cures or specific treatments. Notwithstanding, the employment of facilitatory, albeit aggressive, clinical management approaches has succeeded in enhancing the lifespan and quality of life for patients with these disorders [1,2,3].

Abnormal systemic accumulation of GM1 ganglioside in GM1 gangliosidosis arises from a deficiency of the β-galactosidase enzyme (E.C.3.2.1.23), which hydrolyzes the terminal β-galactosyl residues of this ganglioside, glycoproteins, and glycosaminoglycans [4]. The incidence of GM1 gangliosidosis has been estimated to be 1 in 100,000–2000,000 live births [5], although this pan-ethnic condition has an enhanced prevalence in Roma, Brazilian, Maltese, and Cypriot populations [1,6].

The pathogenic pathway by which such aberrant GM1 storage induces cell death features the build-up of toxic products, which in turn cause an inflammatory response and abnormal mitochondria [7], a mechanism which is common to many neurodegenerative diseases [7]. Moreover, impairments in the regulation of GM1 content are also directly featured in the pathogenesis of Huntington’s and Parkinson’s diseases, and also in a cancer mouse model [8].

Although a developing clinical continuum, GM1 gangliosidosis may be classified into three major sub-strata, which are generally based on the age at which signs and symptoms first appear: infantile, juvenile, and adult (types 1, 2, and 3, respectively). Cases of GM1 gangliosidosis are normally clinically suspected on the basis of the observation of storage signs, including gingival hypertrophy, corneal clouding, coarse facial characteristics, the presence of vacuolated lymphocytes, cherry-red macula, hepatosplenomegaly, and skeletal dysostosis, along with a known history of psychomotor retrogression [1,9,10]. In 2008, Brunetti-Pierri and Scaglia [1] analyzed published cases with sufficient clinical data, and with a diagnosis of GM1 gangliosidosis confirmed by either biochemical assay of β-galactosidase and neuraminidase, and/or by β-galactosidase gene (GLB1) molecular testing. For juvenile (type 2) gangliosidosis, these researchers calculated the incidences of a series of clinical features, and these comprised hypotonia (50%), hypertonia (4%), developmental delay/mental retardation (96%), and seizures (18%) for neurological features; dysmorphic features (67%) for more generalized ones; and cherry-red spot (18%), cardiomyopathy (38%), hepatosplenomegaly (30%), and skeletal abnormalities for the involvement of other systems. However, in the absence of such signs, diagnosis of this condition is rendered difficult or complex [9,11].

Notwithstanding, galactosialidosis represents a genetic disorder which is distinct from GM1 gangliosidosis and its related Morquio B disease, the latter being primarily a skeletal-connective tissue disease, unlike GM1 gangliosidosis which is a neurological condition. In galactosialidosis, the acid β-galactosidase gene is unaffected, but since patients present with deficiencies of both β-galactosidase and sialidase (α-neuraminidase), previously they were frequently incorrectly diagnosed, sometimes as having a GM1 gangliosidosis variant; this again presents diagnostic complications.

In view of the complexity of GM1 disease diagnosis, currently it is mainly determined from the critical assessment of a series of comprehensive observations made on a range of specimen samples collected for biochemical, histological, ultrastructural, and/or genetic analyses. Additionally, such diagnoses may be compromised or complicated by a partial absence or deficit of specimen samples, and consequently this may result in a significant number of cases remaining completely undiagnosed. Hence, there is a major demand for a more disease-specific and expedient protocol for the diagnosis and severity monitoring of GM1 and other debilitating gangliosidosis conditions. With the exception of a small number of selected cases, ganglioside storage diseases are currently untreatable, and hence there is an urgent requirement to seek, test, and validate newly-developed therapeutic strategies. Hence, the identification of novel, clinically-relevant biomarkers (together with associated imbalances in their corresponding metabolic pathways) for the monitoring and prognostic stratification of these diseases and their progression are essential [12]. Although magnetic resonance imaging (MRI) techniques may serve to provide a non-invasive and effective means to monitor disease severity in patients with these conditions [13], this process is expensive and requires onerous, expert-level interpretational skills.

Multicomponent proton (^1^H) NMR analysis of biofluids such as blood plasma, cerebrospinal fluid (CSF), and urine, etc., offers a high level of potential regarding the investigation of metabolic processes, and when coupled with conventional and/or newly-developed multivariate (MV) data analysis techniques, serves as an extremely powerful means of probing and tracking, for example, the biochemical basis of human disease etiology [14,15,16,17,18]. Indeed, this form of combined multianalyte-MV analysis is generally classified as metabolomics, and has been extensively employed in a very wide range of biomedical and clinical investigations, including the identification of diagnostic or prognostic biomarkers for a very wide range of diseases.

In this investigation, we employed an ^1^H NMR-linked metabolomics strategy in order to seek and identify potential new biomarkers of disease activity in blood plasma samples collected from patients suffering from GM1 type 2 (GM1T2, i.e., juvenile classification) disease. Significant metabolic data acquired were then employed to explore GM1 disease-mediated disturbances to metabolic pathways, and also the cellular, organ, or tissue localizations of pathological damage and impairments, using quantitative metabolite set enrichment analysis (QMSEA). In principle, these data may potentially be utilized to (1) provide valuable potential biomarkers for the diagnosis and monitoring of such diseases, and (2) enhance our understanding of disease mechanisms at the biochemical, metabolic, and cellular, organ and tissue levels. Once established in this study, in principle, these biomarkers may be validated in future investigations involving animal models of these diseases which are indeed responsive to therapeutic interventions, since unfortunately, such promising responses are, to date, predominantly absent in human populations. However, it should be noted that very recently, the National Institute of Health (NIH) administered the very first clinical trial for the treatment of GM1 gangliosidosis in humans with a gene therapy (GT), and the manufacturer of the novel adeno-associated virus-based AXO-AAV-GM1 product has received a US Food and Drug Administration (FDA) pediatric disease designation for it [19]. 

In this study, selected drugs and their metabolites were also detectable in the ^1^H NMR profiles of urinary samplings collected from the GM1T2 patient cohort (e.g., the anticonvulsant valproate and its major glucuronide metabolite [20], and/or the muscle relaxant tolperisone, etc.). Therefore, here we have elected to focus on both univariate (UV) and MV metabolomics comparisons of the plasma profiles of GM1T2 patients with those arising from a healthy human control population, since resonances of, or those putatively arising from these xenobiotics and/or their metabolites were not visible in the 700 MHz ^1^H NMR profiles acquired at all. A short account of an experimental pilot study focused on this LSD, which includes a MV power calculation for future studies, has been reported in [21].

## 2. Materials and Methods

### 2.1. Ethical Approval

This investigation was performed in strict accordance with the Declaration of Helsinki 1975 (revised in 2013). All subjects gave their informed consent for inclusion before they participated in the study. The Faculty of Health and Life Sciences Research Ethics Committee, De Montfort University (DMU), Leicester provided ethical approval for the protocol focused on the collection of blood plasma samples from healthy control (HC) participants (reference no. 1936). The GM1T2 patients consented to participate in this investigation under protocol 02-HG-0107 “Neurodegeneration in Glycosphingolipid Storage Disorders” of the National Human Genome Research Institute Institutional Review Board.

Medications received by the GM1T2 patient cohort comprised the substrate inhibitor miglustat (*n* = 1); the anticonvulsant valproate (*n* = 1); the antihypertensive clonidine, which is also suitable for the treatment of a range of other conditions (*n* = 3); lamotrigine for seizures (*n* = 2); the muscle relaxant tolperisone (*n* = 1); the antiepileptic levetiracetam (*n* = 1); orally-inhaled albuterol sulphate for breathing problems (*n* = 1); the anticonvulsant lamotrigine (*n* = 2); the muscarinic antagonist cyclopentolate (*n* = 1); and diazepam for the treatment of seizures and anxiety (*n* = 1).

### 2.2. Collection of Human Blood Samples

Blood specimens were collected from a group of *n* = 10 GM1T2 patients (4 males/6 females, age range 3–20 years (mean 11.4 years)) at the National Institute of Health (NIH), Bethesda, Maryland, USA, and were transported on dry ice to Leicester School of Pharmacy (LSP), Leicester, Leicestershire, LE1 9BH, UK, and then immediately frozen at −80 °C on arrival. Blood was also collected from *n* = 28 HC participants (7 males/21 females, age range 18–54 years (mean 22.4 years)) at LSP. Blood plasma samples were not discolored, and therefore all were included in the analysis. Whole blood was collected into heparinized tubes in order to avoid analytical ^1^H NMR analysis complications arising from the use of those containing ethylenediamine tetra-acetate (EDTA) or alternative anticoagulants such as citrate.

### 2.3. Preparation of Human Blood Plasma Samples for ^1^H NMR Analysis

Blood plasma samples were thawed at ambient temperature and then immediately prepared for ^1^H NMR analysis. This preparation involved centrifuging 500 µL volumes of sample and removing 350 µL volumes of the supernatant for analysis. A 50 µL aliquot of 0.10 M phosphate buffer (pH 7.00) was then added to the clear supernatants, together with a small aliquot of an aqueous sodium azide solution as a microbicidal preservative (final concentration 0.05% (*w*/*v*)), and 10% (*v/v*) ^2^H_2_O. These mixtures were then thoroughly rotamixed, and added to newly-purchased 5-mm diameter NMR tubes ready for high-resolution ^1^H NMR analysis.

### 2.4. ^1^H NMR Analysis of Plasma Samples

Samples were analyzed using a Bruker AVII 700 MHz NMR spectrometer equipped with a ^1^H TCI cyroprobe at an operating frequency of 699,989 MHz and a probe temperature of 298 K. Spectral acquisition involved the collection of 65,536 data points, using 32 scans across a spectral width of 1599 ppm. NOSEY presaturation (noesygppr1d) was used for suppression of the intense water signal located at ca. 4.8 ppm. Moreover, the CPMG pulse sequence was employed in order to suppress the broad protein signal envelope which obscures the visibility of many low-molecular-mass metabolite resonances, and hence also the quantification of their pre-assigned biomolecules. Samples were loaded onto an automated sample belt in a random order.

### 2.5. Preprocessing of ^1^H NMR Biofluid Datasets

^1^H NMR free induction decays (FIDs) were zero-filled by a factor of 2 and multiplied by an exponential function corresponding to 0.30 Hz line broadening prior to Fourier transformation. All spectra were manually-phased and baseline corrected, and chemical shifts referenced to the lactate-CH_3_ function doublet located at δ = 1.330 ppm using Topspin 2.1 (Bruker GmbH, 76187 Karlsruhe, Germany) software. The methyl function doublet resonance of alanine (δ = 1.487 ppm) served as a secondary endogenous chemical shift reference. All spectra were visually examined for errors in baseline correction or referencing, and were then exported to ACD/Labs Spectrus Processor Academic Edition 12.01 (Advanced Chemistry Development, Inc., Toronto, Canada). Intelligent bucketing was applied to all spectra simultaneously with bucket widths of 0.04 ± 0.02 ppm. The intense residual H_2_O/HOD resonance (δ = 4.7–4.9 ppm) was excluded from all spectra acquired. Spectral regions which contained only noise were also excluded from all spectra examined in order to reduce the number of ^1^H NMR bucket variables for metabolomics analysis, and consequently the discriminatory potential of all UV and MV analysis models developed.

Prior to statistical analysis, integral regions of each intelligently-selected bucket (ISB) were constant sum (CS)-normalized, generalized logarithmically (glog)-transformed, and Pareto-scaled. ^1^H NMR spectral resonances were assigned via a consideration of chemical shift values, coupling patterns and coupling constants, and confirmed by reference to literature values and the Human Metabolome Database (HMDB, University of Alberta and The Metabolomics Innovation Centre) [22]. These assignments were further confirmed via the acquisition of two-dimensional (2D) correlation and total correlation spectroscopy (COSY and TOCSY respectively) spectra on 3 or more samples within each disease classification.

Median ^1^H NMR signal-to-noise (STN) ratios were derived from the formula STN = 2.50A/N_pp_, where A is resonance height, and N_pp_ the widest peak-to-peak noise difference determined at each frequency region selected.

### 2.6. Statistical and Computational Intelligence Analysis Strategies Employed

UV, MV, and CIT analyses were performed on two separate models: Model 1 consisted of the full (untargeted) ^1^H NMR dataset containing *n* = 83 ISBs, whereas Model 2 featured a consideration of the intensities of selected key, well-resolved and clearly visible (i.e., targeted) resonances arising from *n* = 25 specified metabolites, i.e., irrespective of their UV statistical significance.

UV analysis-of-variance (ANOVA) of ^1^H NMR ISB intensity data was conducted with *XLSTAT2016 and 2020* software (Addinsoft, Paris, France). In view of clear heterogeneities of the variances (heteroscedasticities) of ISB and specified metabolite variables (Models 1 and 2, respectively) between disease groups (i.e., GM1T2 vs. HC participants) for at least some of these, the robust Welch test was employed to determine statistical significance of differences observed between their mean values. The *p* values of significant variables were then Bonferroni-corrected by multiplying them by the total number of variables considered for both Models. This procedure was adopted in order to maintain a high level of rigor throughout the UV analysis conducted.

Fold-changes were computed as the GM1T2:HC ratios of their mean values for CS-normalized datasets, and where indicated, also for isoleucine (Ile)-normalized ones, since no significant UV differences were found between the HC and GM1T2 classification mean values of this metabolite in this study. These reference metabolite concentration-normalized metabolite level fold-changes were employed for the purpose of comparing their values with those calculated from the mean or median values of an extensive reference dataset of ^1^H NMR-determined blood serum biomolecule concentrations determined on 11–12-year-old childhood and corresponding adulthood (parental) groups in Ref. [23]; the latter comprised calculated child:adult ratios of mean Ile-normalized concentrations. This approach was considered essential in view of significant age differences between this study’s HC and GM1T2 classifications. Hence, any metabolite differences ascribable to participant age differences were readily determined by comparisons of these two sets of reference metabolite-normalized fold-change values. Variances and standard errors (SEMs) for Ile-normalized GM1T2:HC fold-change (mean ratio) indices were computed from a derivation of Taylor’s expansion as described in [24], and 95% confidence intervals (CIs) for these were estimated using the t distribution.

A full Pearson-based correlation analysis was also conducted in order to preliminarily explore inter-relationships between metabolite predictor variables.

Principal component analysis (PCA) was employed to obtain an overview of MV data structure, the degree of separation between/clustering of the different disease classifications explored, and also to detect any potential outliers. This primary PCA approach detected that one of the GM1T2 sampling profiles was a clear outlier, so this was removed prior to further MV analysis. PCA of the Model 2 dataset described below with 25 specified plasma biomolecules was performed both with and without the employment of Varimax rotation and Kaiser normalization, with a maximum of 5 PCs considered (*XLSTAT2016 software*, Addinsoft, Paris, France). Further MV analysis, including additional PCA, and partial least squares- and orthogonal partial least squares-discriminatory analyses (PLS-DA and OPLS-DA, respectively), together with an agglomerative hierarchical clustering (AHC) analysis strategy, was conducted using *MetaboAnalyst v4.0 software* ((University of Alberta and National Research Council, National Institute for Nanotechnology (NINT), Edmonton, AB, Canada) [25]. Successful performance of the PLS-DA and OPLS-DA models were estimated using the leave-one-out cross validation (LOOCV) approach involving a test set (33% of the original number of samples), the remaining 67% being employed to construct the model. Q^2^ and permutation tests were performed to confirm the robustness of such models, the latter with 2000 permutations.

Further permutation testing was also conducted using partial redundancy analysis (P-RDA) of the above CS-normalized, glog-transformed, and Pareto-scaled datasets (*XLSTAT2016*) in order to explore the possible influence of participant ages and genders on ISB explanatory variable intensities. For this purpose, the total number of PCs involved in these models was automatically computationally-determined, and a total of 10,000 permutations were performed for these models. In this manner, the P-RDA technique explored the effects of one out of the three possible model output variables via removal of the influence of the other two as ‘conditioning’ variables’, and therefore this strategy was applied three-fold with either the ‘between-diseases’, ‘between-ages’, and ‘between-gender’ variables serving as the prime prediction output ones for each computation.

The random forest (RF) machine learning algorithm technique was employed for classification and variable selection purposes using the random forest *Metaboanalyst v4.0* module, with 1000 trees (*ntree*) and 7 predictors selected at each node (*mtry*) following tuning. Datasets were randomly split into training and test sets containing approximately two thirds and one third of them respectively. The training set was used to build the RFs model and obtain an out-of-the-bag (OOB) error value in order to assess the performance of the classification. The OOB error term is an estimate of the performance of the RF model (i.e., how often the model classifies a sample incorrectly), and is computed using a test set (one third of the original dataset which is left out of the bootstrap sample used to construct the RF model). The OOB error estimate ranges from 0 (a perfect model where 100% of the test set is correctly classified) to 1 (in which none of the test set was correctly classified). The test set was then used to determine the accuracy, specificity, and sensitivity of this MV analysis strategy. This process was repeated 1000 times in order to prevent bias arising from the random sub-sampling of the training and test sets. The importance of each variable in the classification was determined by computing the average mean decrease in accuracy (MDA) (using the OOB error observations) over all iterations. Discriminatory variables were then ranked in order of importance based on their mean MDA values, and further examination of these values allowed identification of the number of variables required for classification purposes (variables with little or no change in MDA value were defined as redundant and removed).

The support vector machine (SVM) computational intelligence approach was also employed for the purpose of distinguishing between the GM1T2 and HC ^1^H NMR blood plasma profiles, and for this purpose, models were constructed utilizing the above MDA approach. Sequential minimal optimization (SMO) parameters for SVM classification models were C = 1.0 for the regularization criterion, and tolerance and epsilon values of 0.001 and 1 × 10^−12^, respectively; a linear kernel was employed (*XLSTAT2014 software*). SVM results acquired were compared with those arising from the use of sigmoidal, radial basis function (RBF), and power kernels.

For each of these MV data analysis options, model robustness and biomarker reliability were further evaluated using an area under the receiver-operating curve (AUROC) probing analysis, which involved ROC curve generation by Monte Carlo cross-validation, which featured balanced sub-sampling processes using a SVM model builder.

### 2.7. Model Validation

In order to validate results acquired from MV analysis strategies employed, we employed stratified randomized sampling techniques to select a validation set of plasma samples which were used for prediction purposes only.

Two approaches were instigated for validation testing: The first ‘hold-out’ validation set comprised *n* = 6 HC and *n* = 3 GM1T2 samples. Following removal of these sample profiles from the dataset, a secondary PLS-DA strategy, which involved randomly-selected training and validation sets of *n* = 19 and 9 samples, respectively, was utilized, the latter serving as a primary validation checking test set. Subsequently, the accuracy of this model’s ability to successfully predict the classification status of each of the hold-out set of *n* = 9 samples was determined; this process was repeated a total of 72 times, and results are reported for the training, test, and hold-out validation sets separately. This *n* = 9 hold-out validation method was also applied to the AUROC, RF, and SVM biomarker testing techniques conducted.

Secondly, for PLS-DA and all other MV and CIT analysis techniques employed in this study, the degree of predictive classification success of an alternative *n* = 12 hold-out validation sampling set (9 HC and 3 GM1T2 specimens) was determined. Mono- and biclustering AHC-based heatmaps were also generated for the main and hold-out validation sets of both the GM1T2 and HC sample classifications.

### 2.8. QMSEA

**Figure 1:**
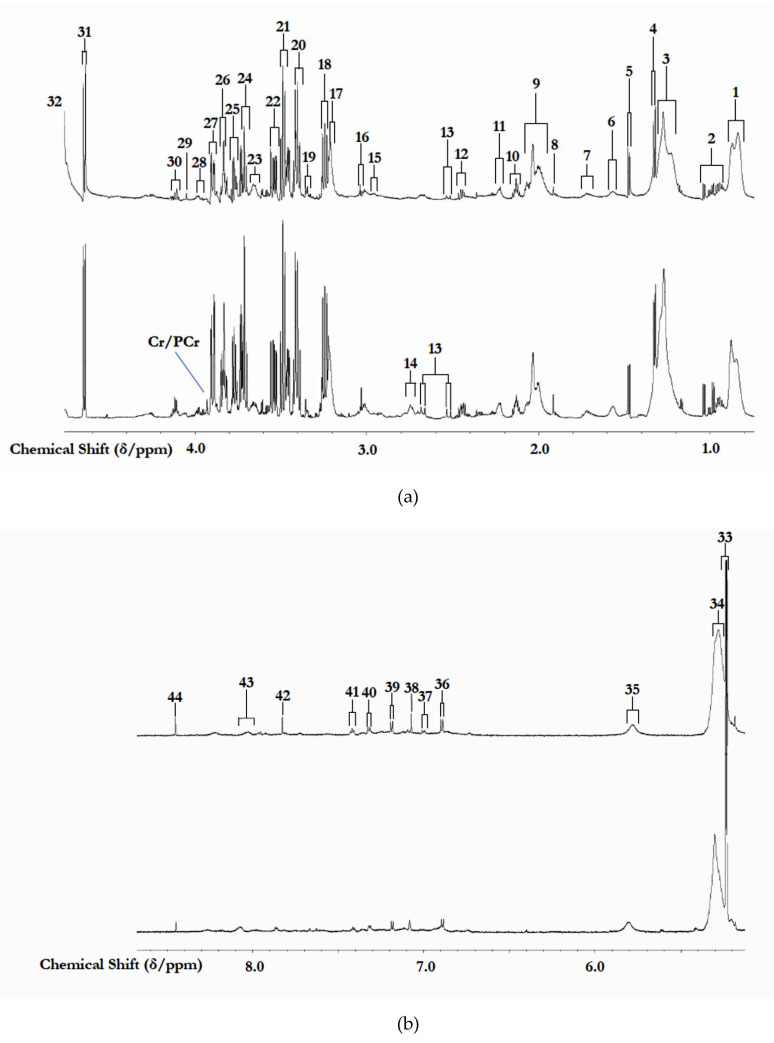
(**a**) and (**b**), expanded 0.75–4.40 and 5.00–8.70 ppm regions, respectively, of 700 MHz CPMG ^1^H NMR profiles of HC (top) and GM1T2 (bottom) plasma samples. Typical spectra are shown. Numerical and abbreviated resonance assignments correspond to the codes provided in Table 1. GM1T2 disease-mediated upregulations in plasma creatinine and lactate concentrations are clearly visible from the intensities of their N-CH_2_- (*s*) and -CH(OH)- (*q*) resonances located at δ = 4.05 and 4.13 ppm (assignments 29 and 30), respectively.

QMSEA was performed on the Model 2 dataset with built-in metabolite set enrichment analysis (MSEA) libraries focused on (1) metabolic pathway-associated metabolite sets (MPAMS) with 99 entries; (2) predicted metabolite sets (PMS) based on a computational enzyme knockout model with 912 entries; and (3) metabolite sets based on estimates for the sub-cellular, cellular, tissue, and organ localizations (73 entries) of disturbed GM1T2 chemopathologies based on the dysfunctional metabolite sets detectable [26]. In view of their complex molecular heterogeneities, biomolecular contributions from the total lipoprotein-associated triacylglycerols (TAGs) (broad triplet, δ centered at 0.92 ppm) and ‘acute-phase’ glycoproteins (broad -NHCOCH_3_ singlet resonances, with δ values of 2.03 and 2.07 ppm), and the broad protein aromatic amino acid residue signal (centered at δ = 8.07 ppm) were excluded from these analyses. The creatine/phosphocreatine (Cr/PCr) signal (δ = 3.95 ppm) was also excluded in view of its heterogeneity, and also possible partial overlap with an intense glucose ring proton resonance (Figure 1). Indeed, only compounds with specified compound names and/or codes recognizable by the software employed were incorporated.

QMSEA was performed using the *globaltest 3* package of *MetaboAnalyst v4.0* [25,26]. This approach employs a generalized linear model to estimate a Q statistic for each metabolite set, which indicates correlations between the profiles of biomolecule levels observed and dysregulated enzymatic functions, metabolic pathways and their human body localization outcomes (these Q statistics for a particular metabolite set represents the mean value of those computed for each metabolite involved).

### 2.9. MV Power Calculations

MV power/minimum sample size estimates were made using the power analysis module of *MetaboAnalyst v4.0* [25]. This preliminary pilot study found that for FDR and overall power levels of 0.05 and 0.80, respectively, a minimum sample size of ca. *n* = 30 per group was required [21]. Although our figure of *n* = 28 for the HC group was almost satisfactory for this purpose, the value of *n* = 10 patients for the GM1T2 cohort (*n* = 9 following outlier removal as described in Section 3.3.1 below) was insufficient. Nevertheless, such experiences are not at all unusual in the LSD field in view of major limitations of biofluid sample availabilities in view of their very low incidences.

## 3. Results

### 3.1. ^1^H NMR Analysis of Study Biofluids Collected

The 700 MHz CPMG ^1^H NMR spectra of these the human blood plasma samples collected contained many prominent, sharp signals assignable to wide range of low-molecular-mass biomolecules; Figure 1 shows the expanded 0.75–4.40 and 5.00–8.70 regions of typical spectra acquired on GM1T2 and HC participants. Indeed, >35 distinct low-molecular-mass metabolites, and the mobile portions of biomacromolecules, were detectable in the CPMG spectra acquired on this biofluid, and these included short-chain organic acid anions (e.g., acetate, formate, citrate, and lactate, etc.); amino acids, including leucine, Ile and valine (branched-chain amino acids), glycine, alanine, glutamate, glutamine, lysine, taurine, histidine, phenylalanine and tyrosine, etc., together with *N*-acetylamino acids; and carbohydrates, most especially glucose (Figure 1). As expected, these ^1^H NMR profiles also contained relatively broad resonances arising from a series of lipoprotein-associated TAGs, with ^1^H NMR-distinguishable very low-, low-, and high-density-lipoproteins; the acetamido (-NHCOCH_3_) functions of *N*-acetylneuraminate and *N*-acetylglucosamine residues present in the molecularly-mobile carbohydrate side-chains of selected ‘acute-phase’ glycoproteins (predominantly α-1-acid glycoprotein); and those arising from both aliphatic and aromatic amino acid protein residues. A full list of ^1^H NMR assignments for biomolecules detectable is provided in Table 1. This table also provides median STN values for all resonances present.

### 3.2. Univariate and Preliminary Metabolomics Analysis of Biofluid ^1^H NMR Profile Datasets

**Figure 2:**
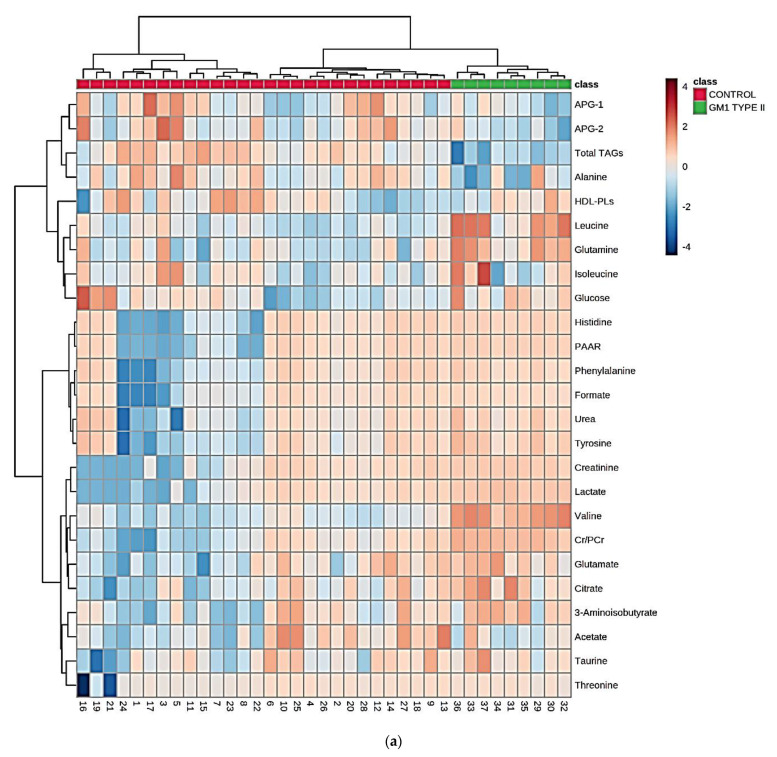

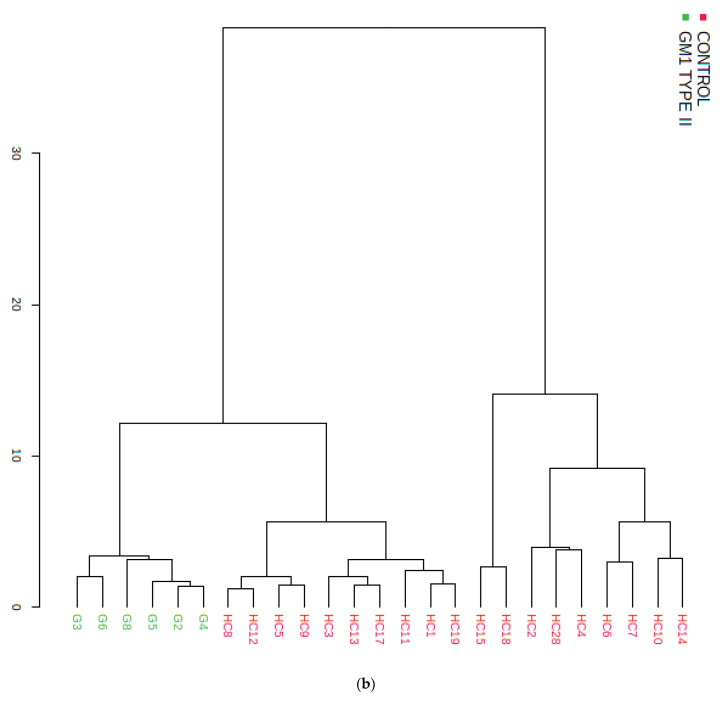

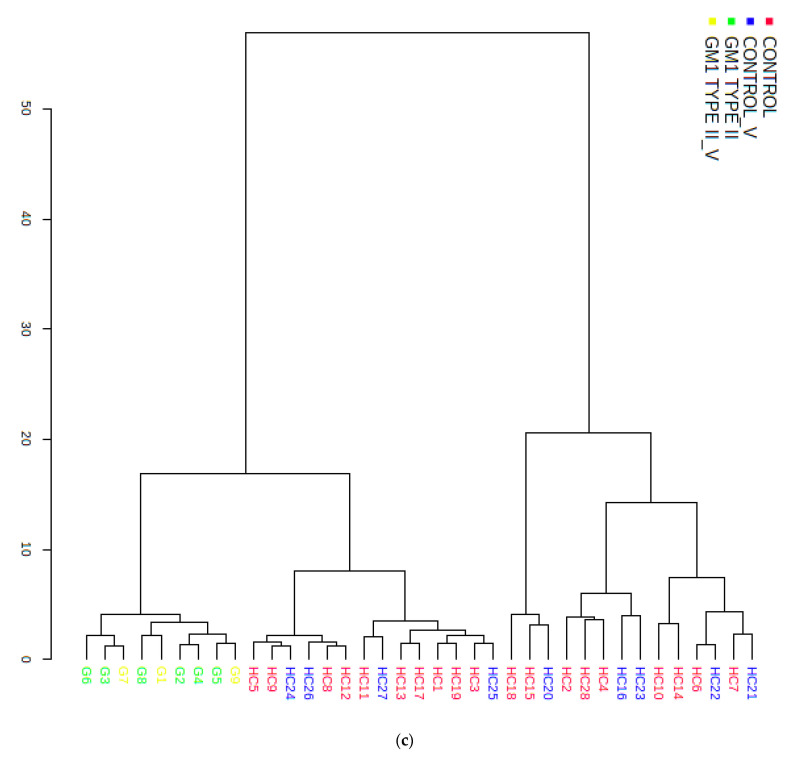

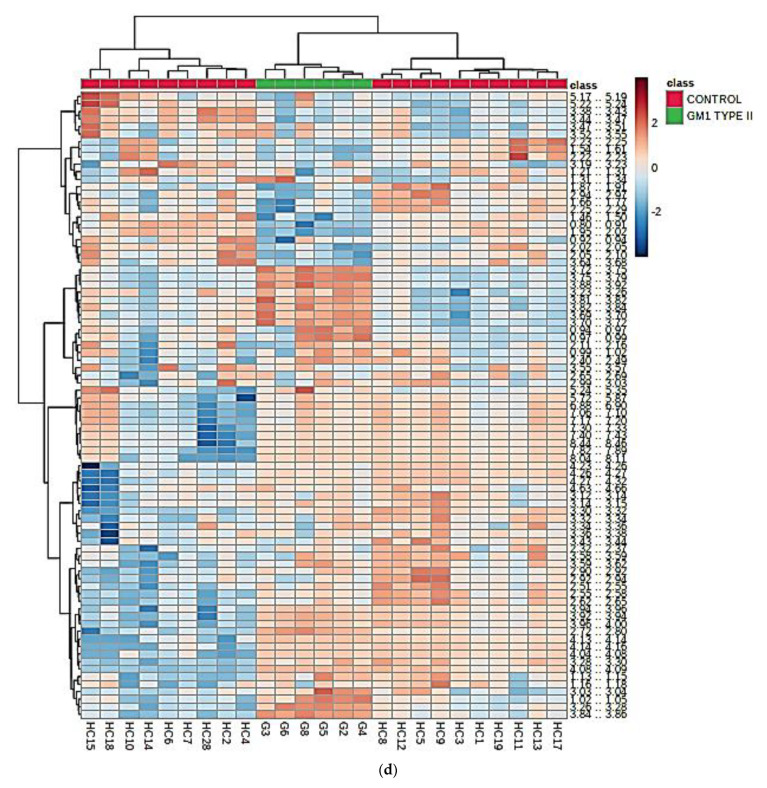

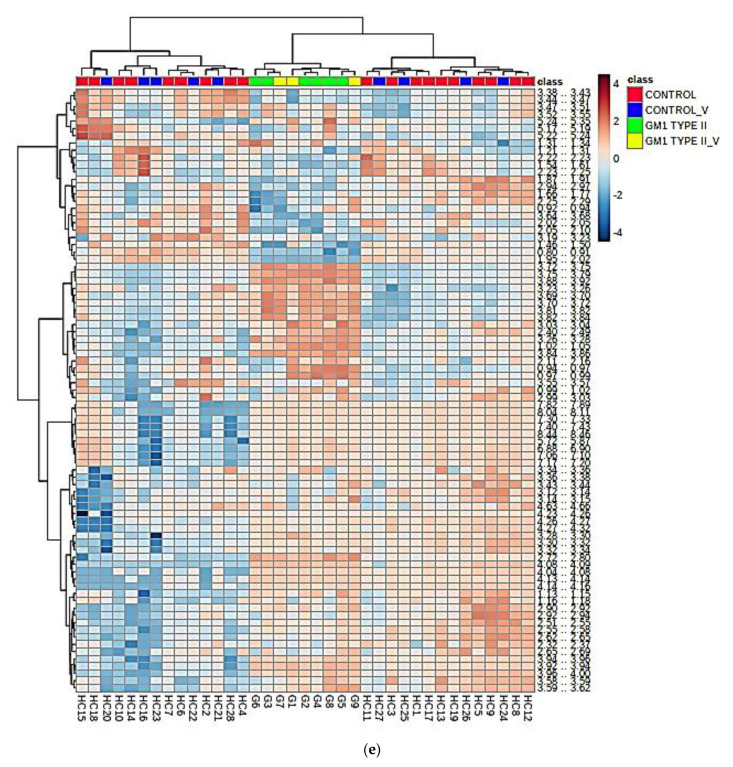
(**a**) AHC biclustering heatmap diagram displaying the most univariately-significant differences between the ^1^H NMR profile predictor metabolite variables of the GM1T2 (green) and HC (red) groups investigated (Model 2 dataset); those for all 25 variables are shown (near right-hand side *y*-axis).^1^H NMR datasets were CS-normalized, glog-transformed, and Pareto-scaled prior to analysis. Transformed metabolite ^1^H NMR intensities are shown in the far right-hand side *y*-axis: deep blue and red colorations represent extremes of low and high concentrations, respectively. The left-hand side of the plot shows results arising from an AHC analysis of the top 25 metabolite variables monitored, which reveals 2 major metabolite clusterings, with 2 sub-clusterings within each of these. (**b**) AHC dendogram of a ‘training’ set consisting of *n* = 19 HC and *n* = 6 GM1T2 disease participant sample donors, and revealing clearly distinctive MV clusterings between the blood plasma ^1^H NMR profiles of these groups for the Model 1 ^1^H NMR ISB dataset. (**c**) As (**b**), but with inclusion of the hold-out validation sampling set of *n* = 9 HC and *n* = 3 GM1T2 samples. (**d**) AHC biclustering heatmap diagram performed on the ‘training set’ of 19 HC and 6 GM1T2 for the Model 1 dataset. (**e**) As (**d**), but with incorporation of the hold-out validation sampling set. AHC models applied involved a consideration of maximum dissimilarities from Euclidean distances. Abbreviations: CONTROL_V and GM1T2_V represent samples of the hold-out validation set (blue and yellow color-coded labels, respectively).

A rigorous univariate, false discovery rate (Bonferroni)-corrected ANOVA Welch test (WT) system was employed to primarily examine the univariate statistical significance of ‘between-disease’ differences in the mean resonance intensities of CS-normalized, glog-transformed, and Pareto-scaled ^1^H NMR-detectable metabolites. For the uncorrected WT, mean differences between 20 of the biomolecules detectable were found to be significant or highly significant (*p* values ranging from 10^−5^ to 0.04, Table 2). However, application of the additional Bonferroni adjustment to these WT statistic values narrowed the number of significant biomolecules to 13. Figure 2a displays a heatmap of the top 25 specified ^1^H NMR-identified metabolite variables arising from this analysis, and this clearly confirms significantly higher plasma levels for 12 or more of them, and lower plasma levels for TAGs and alanine, in the GM1T2 group of blood plasma samples analyzed. These data clearly demonstrated that, in a univariate context, plasma valine, glutamine, glutamate, citrate, creatinine (Cn), lactate, urea, tyrosine, phenylalanine, and histidine were significantly or highly significantly upregulated in the GM1T2 patient cohort, whereas total plasma lipoprotein-associated TAGs were significantly downregulated in this group.

Monoclustering AHC analysis shown in the form of heatmaps demonstrated 2 major metabolite variable clusterings, with 2 sub-clusterings within each of these. The first and second sub-clusters within the top left-hand side ordinate axis cluster feature (1) acute-phase glycoprotein/TAG-CH_2_-CH=CH- signals 1 and 2, total lipoprotein TAGs, and alanine (all downregulated with respect to GM1T2 disease), and (2) leucine, Ile, glutamine, and glucose (all upregulated); sub-clusters within the bottom left-hand side ordinate axis cluster incorporated (1) all upregulated aromatic amino acids (phenylalanine, tyrosine, histidine, and the PAAR signal), along with urea and formate, and (2) Cn, lactate, valine, Cr/PCr, glutamate, citrate, 3-aminoisobutyrate (3-AIB), acetate, taurine, and threonine (mainly also upregulated predictors).

The biclustering heatmap approach applied to the full sample dataset of Model 2 (Figure 2a) demonstrated that the top left-hand side metabolite APG-1 and −2/total TAGs/alanine (all downregulated) sub-cluster, and the leucine/Ile/glutamine/glucose/HDL-phospholipid (all upregulated) sub-cluster were strongly associated with GM1T2 disease. Similarly, further GM1T2-linked sub-clusters were those containing all aromatic amino acids, the protein aromatic amino acid residue resonance, formate, and urea (all upregulated); that containing Cn, Cr/PCr, lactate, valine, glutamate, and citrate (all upregulated); and less powerfully so, that comprising 3-AIB(↑), acetate(↓), taurine(↑) and threonine(↑), although no significant differences were noted for acetate and threonine.

Figure 2b,c shows AHC-based mono-clustering analyses of the training set (*n* = 26 in total), both with and without inclusion of the hold-out validation HC and GM1T2 classification sub-groups, respectively. In addition to confirming a very high level of distinctiveness between the HC and GMIT2 disease cohorts, these results demonstrated a 100% rate of classification success for the hold-out samples. Morerover, Model 1 biclustering heatmaps for the training, and training plus hold-out validation sets, are shown in Figure 2e,f, respectively, and the ^1^H NMR ISBs featured correspond to the up- and downregulated plasma metabolites tracked in Model 2, for example BCAAs, Cn, lactate, glucose, urea, aromatic amino acids, etc., for the former, and alanine and lipoprotein-associated TAGs for the latter (assignments for these ISBs are available in Table 1). As expected, many of the smaller clusterings detectable correspond to highly-correlated resonances arising from the same molecular sources, typically those of glucose C2-H to C5-H ring protons (δ = 3.2–3.9 ppm), lipoprotein-linked TAGs (δ = 0.9–5.4 ppm), and the ring protons of aromatic amino acids (δ = 6.8–8.1 ppm), etc. (Table 1). Notwithstanding, all 12 hold-out validation samples analyzed were correctly located within their known identity clusters, i.e., the central GM1T2 one, and those from the HC population either side of it. Curiously, the GM1T2 group is sub-clustered with the right-hand-side HC one, and hence it appears that the distinct metabolic patterns identified for the former have a degree of similarity to those of the latter group.

However, on consideration of differences between the mean ages of the GM1T2 and HC cohorts recruited to this investigation, along with complications associated with the recruitment of a large-sized healthy control group of infants, juveniles, and young adults, an already available ^1^H NMR-determined blood serum metabolite concentration dataset was referenced to enable direct age-matched comparisons to be made between the ^1^H NMR-determined blood serum metabolite levels of healthy children accessible therein, and our GM1T2 group (age range 3–20 years). This full and very extensive dataset, extracted from Ellul et al. (2019) [23], provided mean±SD blood serum metabolite concentrations on a total of *n* = 1170–1180 child participants aged 11–12 years; this limited age range is very similar to both the mean and median ages (11.1 and 8.0 years, respectively) of our GM1T2 participants. In this report [23], corresponding metabolite levels were also provided for the child participants’ parental adult ‘controls’ (*n* = 1310–1320). Non-normalized child:adult fold-change values derived from the ratios of mean metabolite levels in the child group to those of the adult classification of Ref. [23] are provided in Table 2. Although these data provide evidence for some GM1T2 disease-upregulated amino acids, creatinine, lactate and urea, along with downregulated lipoprotein-associated TAGs, these are not strictly comparable since CS normalization was employed in this study to provide normalized resonance ^1^H NMR intensity measurements for statistical analysis (which were also generalized logarithmically (glog)-transformed and Pareto-scaled prior to its instigation).

Therefore, Ile was selected as an alternative normalization feature as outlined in Section 2.6. In this manner, statistical evaluations, both univariate and multivariate, were performed on the Ile-normalized dataset in order to allow comparisons with a similarly-normalized child dataset obtained from Ref. [23]. Ile normalization was performed by expressing the raw intensities of both Model 1 and 2 ^1^H NMR resonance bucket intensities to that of the Ile-CH_3_ proton resonance (*d*, δ = 0.997 ppm) in spectra acquired on the GM1T2 and HC datasets. Corresponding serum Ile concentrations available in the childhood dataset of Ref. [23] were similarly employed as normalization vectors for all other ^1^H NMR-detectable metabolites determined in that study. Metabolites available from the latter source comprised total triacylglycerols/fatty acids, total phospholipids and cholines, acute-phase glycoproteins (predominantly α_1_-acid glycoprotein), acetate, citrate, lactate, and creatinine, along with the amino acids Ile, leucine, valine, glutamine, phenylalanine, tyrosine, and histidine. Primarily, comparisons of the Ile-normalized metabolite concentrations of our GM1T2 patient cohort with those of our study’s more adult HC group were then performed (Table 3). This analysis revealed that 15 of these biomolecules were significantly different between these groups when using the WT, and following Bonferroni correction, 8 of these remained so, specifically downregulated total TAGs and acute-phase glycoprotein signal 2, together with upregulated valine, tyrosine, histidine, the detectable aromatic amino acid protein residue, creatinine, and lactate levels.

Subsequently, both GM1T2:HC (this study) and child:adult (Ref. [23] study) fold-changes (i.e., as ratios of the Ile-normalized mean metabolite concentrations for each of these biomolecules) were calculated, and these are also available in Table 3. From the 95% CIs computed for this investigation’s Ile-normalized metabolite level GM1T2:HC fold-changes, it was found that those for lactate > creatinine > valine > histidine > tyrosine > phenylalanine were significantly greater than corresponding age-based fold-changes derived from study [23], in that order. However, apert from citrate, those for TAGs and alanine were found not to be significantly lower than the latter age-based fold-change values. Notwithstanding, despite the unavailability of comparator data in Ref. [23], the Ile-normalized mean concentration GM1T2:HC fold-changes of 3-AIB, urea, PAAR, and formate remained significantly greater than the reference null hypothesis value of 1.00 used for this comparison.

### 3.3. Multivariate Metabolomics Analysis of Biofluid ^1^H NMR Profile Datasets

#### 3.3.1. PCA, PLS-DA, and OPLS-DA

**Figure 3:**
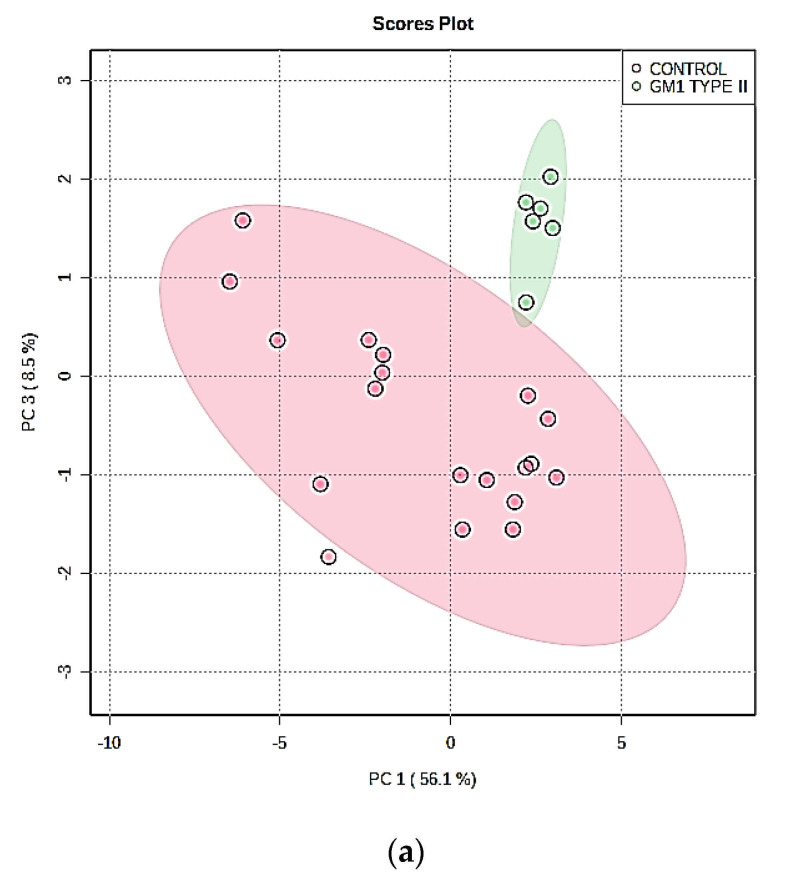

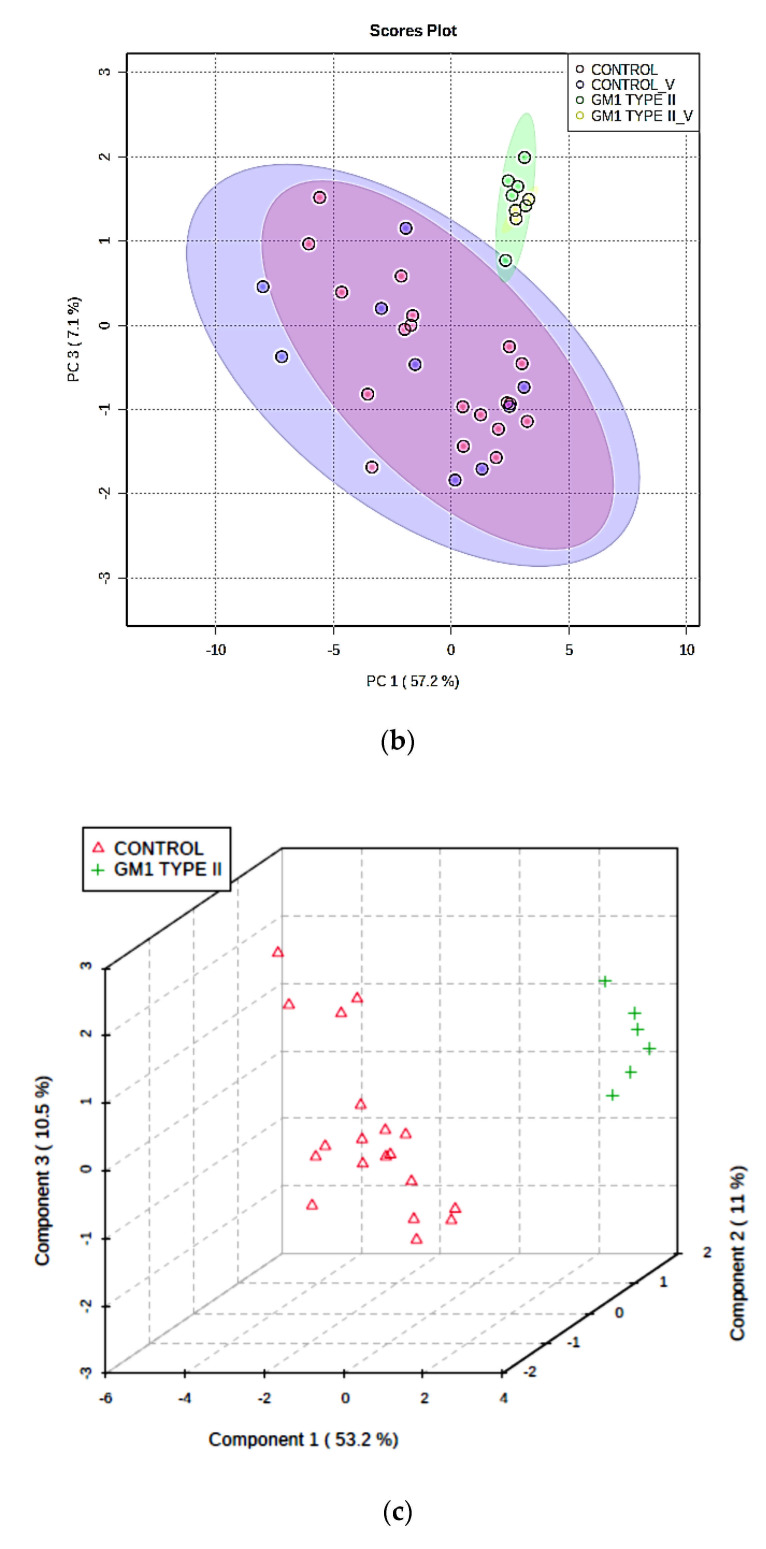

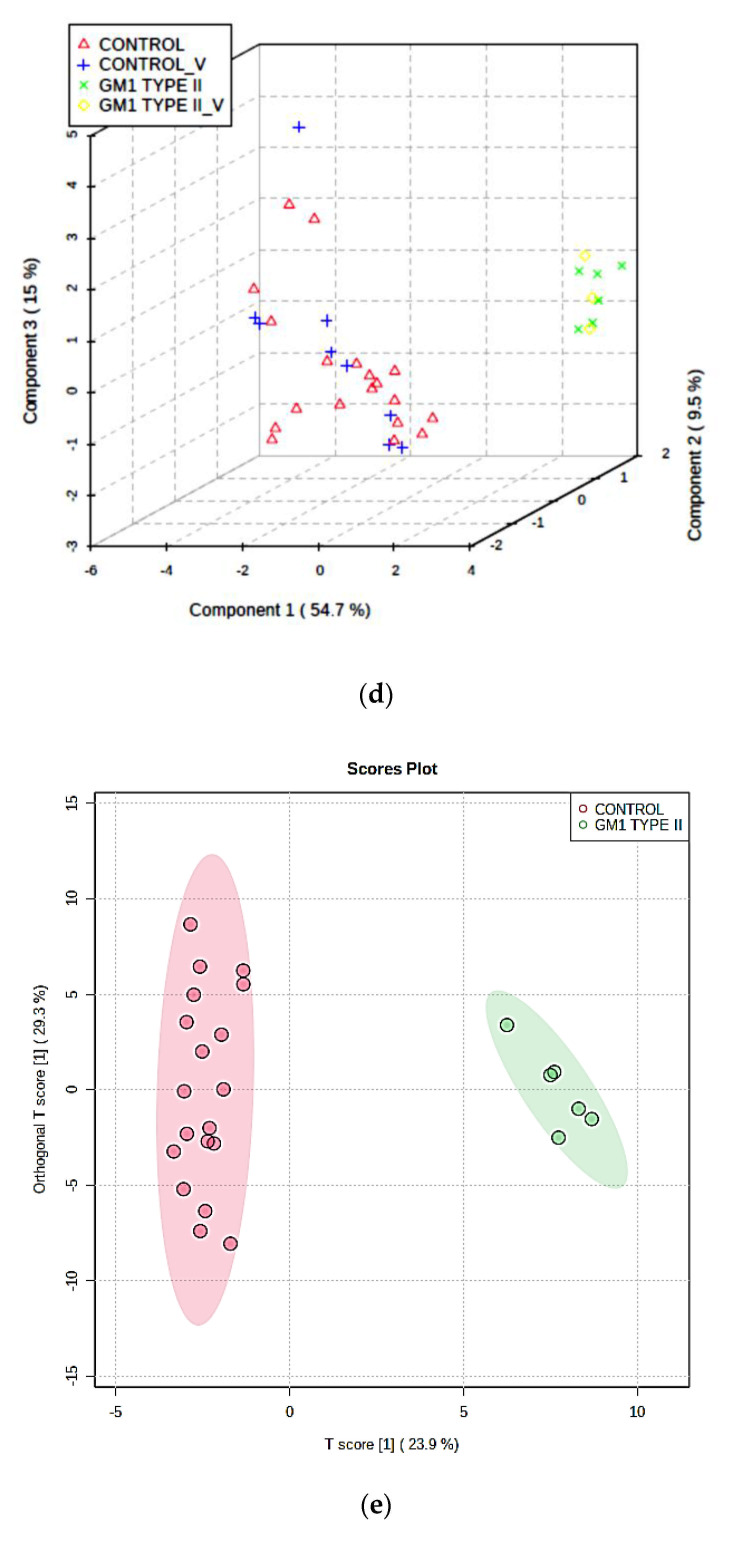

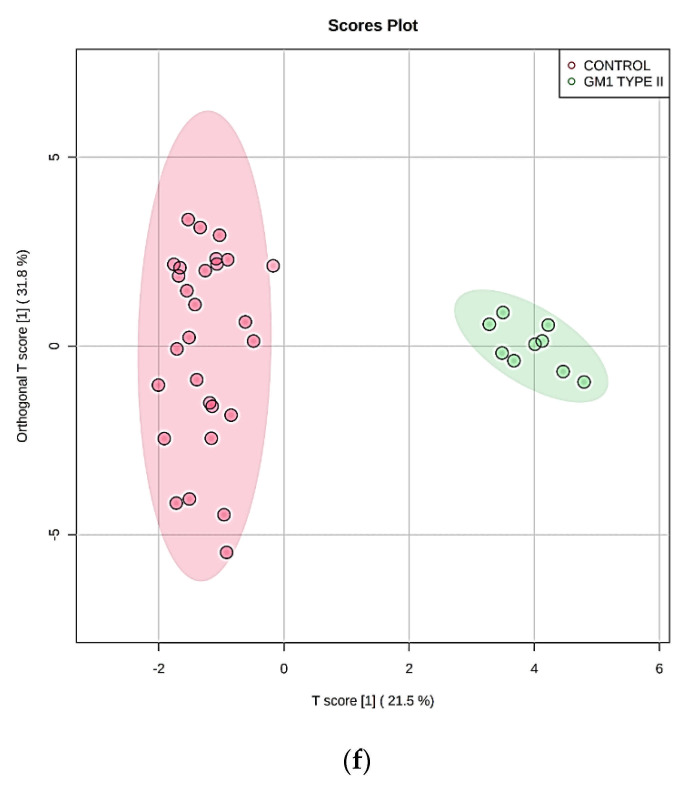
(**a**,**b**). PCA scores plot of PC3 *versus* PC1 for the training (*n* = 25), and training plus ‘hold-out’ validation (*n* = 37 in total) Model 1 datasets, respectively (in (**b**), PC1 and PC3 accounted for 57.2 and 7.1% of total dataset variation respectively). These plots clearly show distinctive clusterings for the HC (red) and GM1T2 patient (green) study classification participants, and (**b**) confirms correct classifications for all hold-out samples evaluated. (**c**,**d**). Three-dimensional (3D) PLS-DA scores plots of component 3 vs. component 2 vs. component 1 for the training, and training plus hold-out validation, Model 1 datasets, respectively. (**e**,**f**). OPLS-DA plot of orthogonal T score [1] versus T score [1] for the Model 1 training, and complete ^1^H NMR plasma profile dataset, respectively (the latter including the *n* = 12 validation set held out from the analysis shown in the former), again revealing clear distinctions between the HC and GM1T2 participant clusters. 95% Confidence ellipsoids are also shown for both participant classifications and their validation sub-sets.

Unsupervised PCA was primarily performed on the Model 1 dataset, and following the removal of a single outlier sample, this MV approach demonstrated very clear distinctive clusterings for each of the two disease classifications (Figure 3a,b), and this proved that this MV analysis technique was efficient for their distinction with the ^1^H NMR-bucketed dataset acquired. It was also found to be very effective for the accurate classification of a hold-out sub-set of *n* = 12 plasma samples (Figure 3b). Similar results were obtained for Model 2, and for this analysis, communalities of metabolite predictor variables were determined from their loadings vectors on principal components (PCs) 1–5. Indeed, these values and their squared cosine values (Table 4) indicated that of the 25 individual biomolecular predictors considered, all aromatic amino acids, along with formate and urea, all loaded very strongly and positively on PC1; all branched chain amino acids (BCAAs) loaded significantly and positively on PC2 (this observation is borne out by the quantitative metabolite enrichment analysis (Section 3.5), which revealed that imbalances in BCAA catabolism were a major feature of GM1T2 chemopathology); the powerful discriminatory biomarkers lactate and Cn loaded strongly and positively on PC3, i.e., their plasma levels were strongly correlated; a combination of the macromolecular APG-I, APG-II, and HDL-PL biomolecules exerted powerful contributions towards PC4, the first two positively, and the latter negatively so; and 3-AIB and alanine loaded strongly (positively and negatively so respectively) on PC5, with positive contributions from acetate and citrate also loading moderately so (however, it should be noted that this PC’s eigenvalue was only 1.05). The total TAG variable loaded strongly and negatively on both PC1 and PC2 respectively, these anticorrelations being stronger with the latter. As may be expected, acetate, a metabolite associated with fatty acid (FA) biosynthesis, also loaded significantly on PC1, but positively so. Moreover, the Cr/PCr signal positively correlated with that of their downstream Cn metabolite, with strong loadings on PCs 1 and 3; this is consistent with their contiguous metabolic pathway(s).

Subsequently, PLS-DA was employed to explore the ability of this technique to distinguish between the GM1T2 and HC blood plasma ^1^H NMR profiles acquired. Figure 3c,d displays 3D PLS-DA scores plots for the Model 2 dataset, and these demonstrate clearly distinctive clusterings for these two groups using the CS-normalized dataset, and also a very successful classification performance accuracy with the *n* = 12 hold-out dataset. To evaluate the performance of this MV classification system, a 10-fold cross-validation procedure was applied. Q^2^ values determined for this analytical model were >0.93 and 0.80 for Models 1 and 2, respectively (both analytical strategies containing a maximum of ≥5 orthogonal components); these statistics were very highly significant, since values of this index of ≥ 0.50 are commonly utilized as a model merit cut-off for this system [17]. Moreover, permutation tests for Q^2^ (with 2000 permutations) yielded *p* values of <0.01 and 0.007 respectively for these MV approaches. PLS-DA variable importance parameters (VIPs) were employed to identify the most important metabolite predictor variables for distinguishing between these two groups of participants, and in addition to all those detectable from the univariate ANOVA Welch test, for Model 2, formate (GM1T2-upregulated) was found to serve as a significant NMR-detectable biomarker feature. For this dataset, the highest VIP values obtained for these distinguishing biomarkers were lactate (2.09) PPAR (1.91) > Cn (1.72) Cr/PCr (1.53) > histidine (1.48) > valine (1.42) > formate (1.17) > urea (0.98) > phenylalanine (0.94) > tyrosine (0.92) > leucine (0.85) > total lipoprotein-associated TAGs (0.85).

Similarly, application of the OPLS-DA technique also confirmed a high level of distinction between the GM1T2 and HC groups for the full dataset of Model 1, with a very clear distinction between them (Figure 3e,f). Indeed, this distinctiveness was also found on application of this technique to the *n* = 25 training set alone, and also correctly classified all 12 of the hold-out validation samples. Key discriminatory ISB variables for this MV analysis strategy were determined from the extremes of its associated characteristic p(corr)[1] versus p [1] S-plot. These included valine, leucine, glutamine, lactate, Cn, citrate, and histidine at the top right-hand-side of this S-plot (all upregulated in GM1T2 diseases), and total lipoprotein TAGs and alanine at the bottom left-hand-side of it (both downregulated). The Q^2^ value computed from an analysis of the complete Model 1 dataset was 0.55, and the permutation statistic computed for it had a very highly significant *p* value of <5 × 10^−4^. As expected, a repetitive application of the above MV analysis strategies to the Ile-normalized Model 2 dataset also provided evidence for a high level of distinctiveness between the HC and GM1T2 disease groups. For example, PLS-DA yielded Q^2^ values of >0.80 for models with ≥4 components, together with a permutation testing statistic *p* value of 0.007. Similarly, a corresponding OPLS-DA MV strategy gave rise to an effective classification model with Q^2^ and R^2^Y values of 0.483 and 0.563 (data not shown).

#### 3.3.2. Supporting Model Validation and Predictive Accuracy Estimates

In addition to employing the *n* = 12 hold-out validation set described above, application of an alternative validation method with *n* = 9 hold-out samples, specifically 6 HC and 3 GM1T2 ones (Section 2.7), provided further supporting evidence for the effectiveness and predictive classification accuracies of the MV analysis approaches described above. This approach, which primarily involved application of the PLS-DA strategy to the CS-normalized dataset, found that the total successful classification rate for this *n* = 9 validation sample set was >97%; those for the ‘training’ and ‘test’ sets were ca. 98 and 95% respectively (Table 5).

#### 3.3.3. RF and SVM Classification Strategies

The RF and linear kernel-based SVM computational intelligence analysis strategies applied displayed very high accuracies for predicting these disease group classifications. Indeed, these were 98 and 100% overall for RF analysis performed on CS-normalized Models 1 and 2 datasets respectively (with all 27 HC and 8 out of 9 GM1T2 samples correctly classified for the former). Key biomarkers found with this RF approach were valine > Cr/PCr > lactate > total triacylglycerols > leucine > Cn > glutamine > PAAR > histidine > alanine in that order of importance. Similarly, these classification values were 94.0 and 98.3% for SVM models with totals of 17 and 29 ^1^H NMR ISB variables incorporated for Model 1, and ≥99% for Model 2 with 10 or more predictor variables incorporated (data not shown).

However, performance of the SVM analysis with independent training/test (*n* = 28) and *n* = 9 hold-out validation sets yielded outstanding classification results when evaluating the Model 2 dataset, i.e., 100% correct classification successes. Indeed, 12 repeats of a SVM application involving *n* =22 training, *n* = 6 randomly-selected test, and independent *n* = 9 hold-out validation sets confirmed this 100% success rate for all sub-set classes, However, application of SVM strategies involving sigmoidal, RBF, and power kernels were all less effective at successful classification, with average error rates observed ranging from 1 to 8%.

#### 3.3.4. AUROC Testings of Complete and Hold-out Validation Datasets

**Figure 4:**
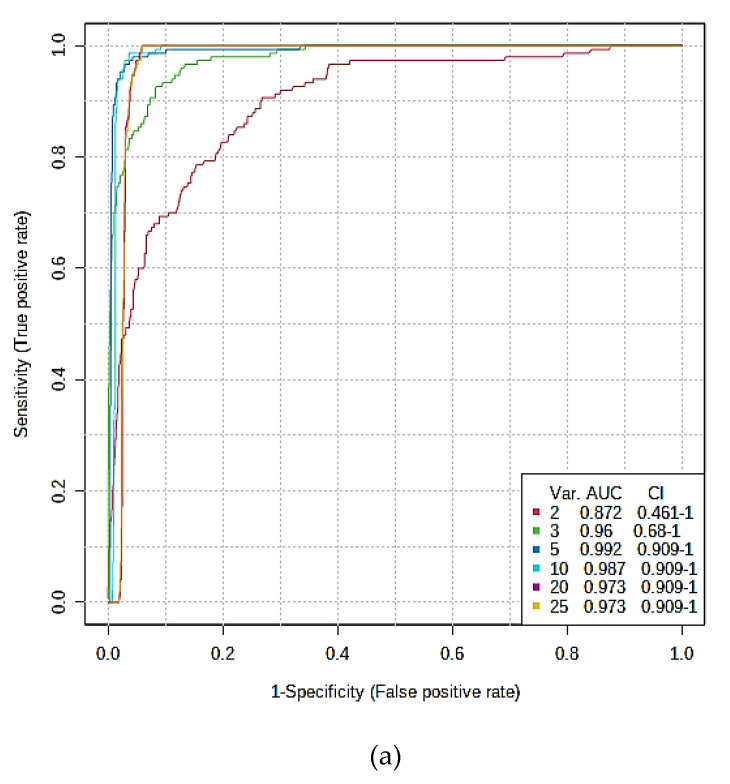

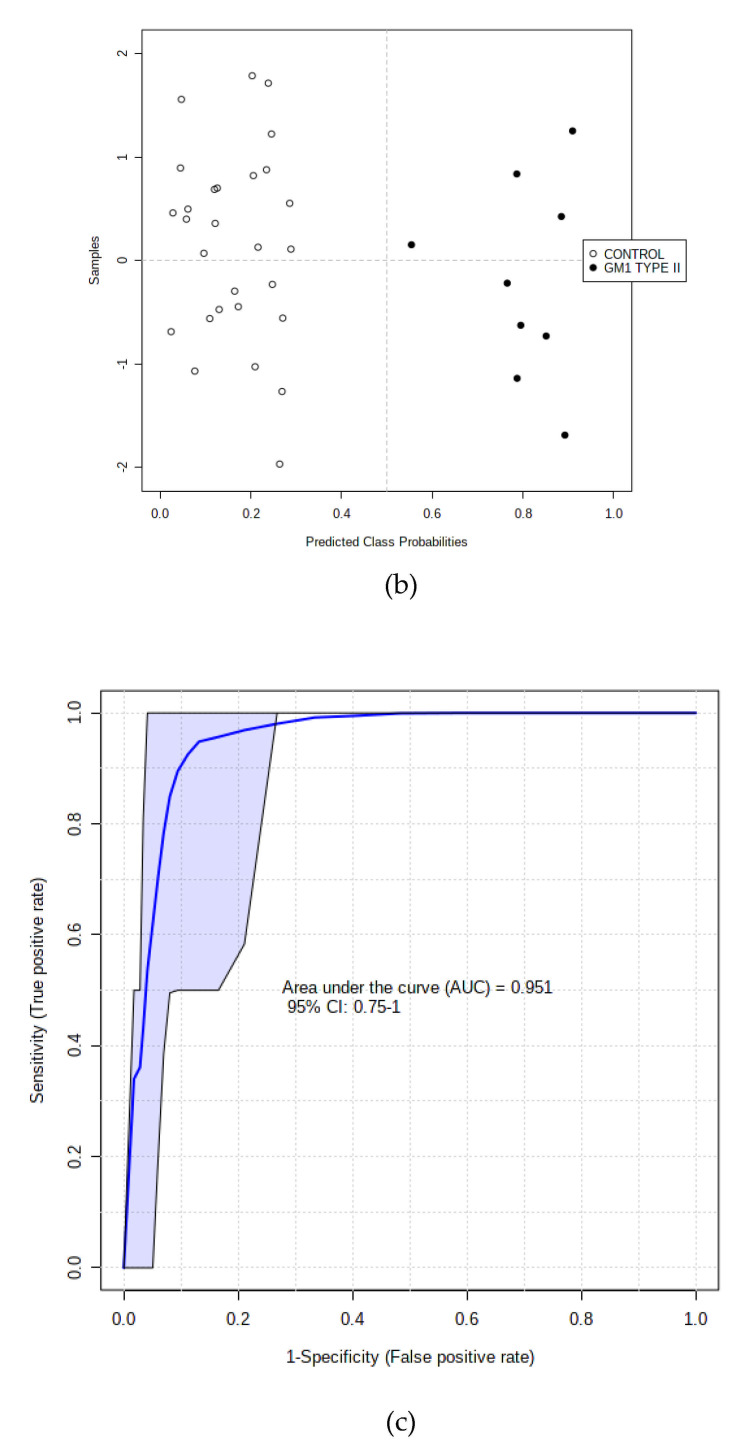
(**a**) Receiver-operating characteristic (ROC) curves arising from a linear kernel SVM-based cross-validation analysis of the Model 1 ISB-containing dataset for systems with 2–25 variables. (**b**) Predicted class probability plot for the most effective system for Model 1 featuring 10 predictor variables (all samples were correctly classified). (**c**) Corresponding ROC curve for analysis of the Model 2 dataset, showing the best system with only 5 predictors; 95% CIs are also shown.

Validated and cross-validated determinations of AUROC values were selected as MV methods to augment the MV analysis approaches employed above, specifically to evaluate the reliability of biomolecular distinctions between the two sampling groups explored. Using a linear kernel SVM strategy, estimates of this methods’ accuracies were then made from validated AUROC indices, which were 0.978 (0.80–1.00), 0.998 (0.985–1.00) 1.00 (1.00–1.00) and 1.00 (1.00–1.00) for systems with 3, 5, 10, and 20 variables, respectively, considered for the Model 1 dataset (95% CIs in brackets), an observation confirming the efficacy of this MV testing strategy (Figure 4a,b).

Moreover, application of this approach to the Model 2 dataset yielded corresponding AUROC values (95% CIs) of 0.872 (0.46–1.00), 0.960 (0.68–1.00), 0.992 (0.91–1.00), and 0.987 (0.91–1.00) for systems with 2, 3, 5, and 10 predictor variables considered, respectively, for the full dataset (Figure 4c); strategies with 20 and 23 variables incorporated were less reliable than that with only 5 predictors (although both had 95% CIs of 0.91–1.00), and therefore those containing 5 predictors only were more than sufficient for distinguishing the GM1T2 plasma profiles from those of the HC participants. In this case, the order of effectiveness of these top predictor biomarker variables (with rank frequencies in brackets) were valine (0.98) > acetate (0.88) > leucine (0.68) > total TAGs (0.48) > HDL-PLs (0.40).

Further testing of the Model 2 dataset involved the *n* = 9 sample hold-out validation strategy noted above in Section 3.3.2, and this system was found to have a successful classification rate of 22/22 HC and 6/6 GM1T2 disease for the training set. Moreover, a high degree of classification effectiveness for the 9 hold-out validation test samples (6 HC and 3 GM1T2 plasmas) was also achieved. The mean accuracy based on 100 cross-validations was 0.897 (1000 permutation test *p* value < 0.001).

### 3.4. Further Investigation of Potential Metabolic Influences Exerted by Participant Ages and Genders

Permutation testing via P-RDA was adopted as a further MV analysis strategy in order to evaluate the potential effects exerted by the ages and genders of participants ‘nested’ within the two disease classification explanatory variable (when determining the significance of either the disease group, participant age, and gender variables, the statistical significance of the considered variable was tested with the other two serving as ‘conditioning’ ones). These analyses demonstrated that whilst the ‘between-diseases’ effect was indeed highly significant (*p* = 4.80 × 10^−3^), as expected, those for the age and gender factors were not (*p* = 0.46 and 0.79, respectively). Therefore, there was no evidence available for the significant contribution of the age and gender demographic variables towards the variance of any of the ^1^H NMR ISBs investigated.

Additional investigations of the potential influence of the age variable were explored by simple Pearson correlation and PCA analysis. For Model 2, only weak linear correlations were found between total TAGs (r = 0.56), leucine (−0.50), valine (−0.57), alanine (0.48), and glutamine (−0.38) and participant age; however, these were rendered insignificant when these were FDR-corrected. Moreover, a PCA model incorporating participant age as a separate possible predictor variable revealed that it only loaded weakly on all of the first 4 plasma metabolite PCs evaluated (squared cosine values of loading vectors 0.006–0.25), and this again indicated little or no contributions of the age variable towards variability of metabolite variables. Interestingly, an increase in plasma TAG concentrations with increasing age may be expected, and therefore the positive correlation observed between them may indicate this. Moreover, data available from Ref. [23] demonstrated that the mean child:adult fold-change of total TAGs and total fatty acids were 0.808 and 0.842, respectively (Table 2).

### 3.5. Quantitative Pathway Enrichment Analysis of Significant Biomolecules

**Figure 5:**
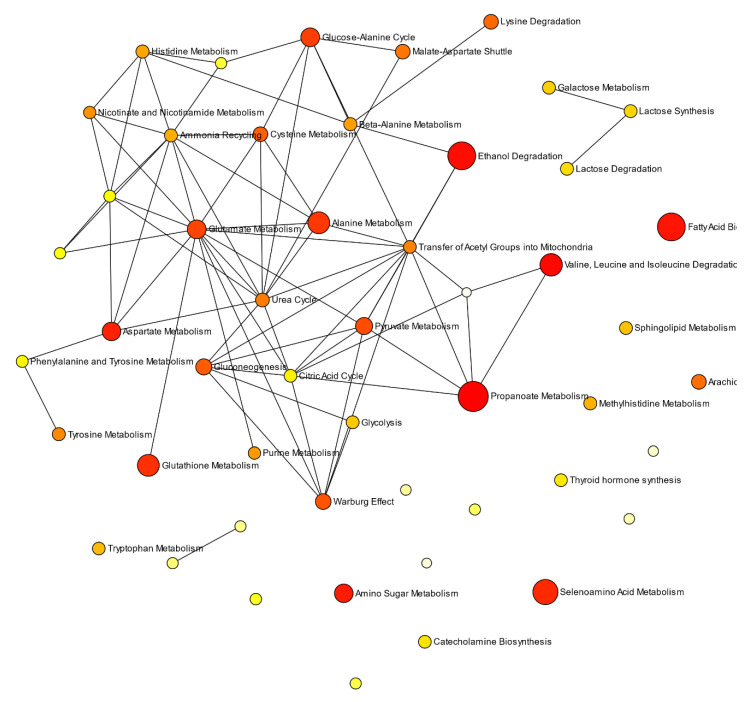
QMSEA metabolic pathway network analysis of GM1T2-mediated disturbances to plasma biomolecule patterns with *MetaboAnalyst v4.0*. This analysis shows dysregulated metabolic pathways found, and interactions/connectivities between them. The statistical significance of imbalanced pathway features decreases in the color code order red > orange > yellow, and their overall GM1T2 disease impact is reflected by their radii, with the most affected ones being denoted by large values, the least affected by small ones.

Firstly, quantitative MPAMS bioinformatics analysis of the Model 2 dataset demonstrated that GM1T2 disease-induced disturbances in propanoate metabolism, BCAA and ethanol degradation, FA biosynthesis, and amino-sugar and aspartate metabolism were the most highly significant, with FDR-adjusted *p* values ranging from <10^−11^ (propanoate metabolism) to <10^−4^ (aspartate metabolism), as listed in Table 6. Therefore, this provides evidence that these pathways represent significant features of the GM1T2 disease classification, when comparatively evaluated against the HC group. Changes in seleno-amino acid, glutathione (GSH), and alanine metabolism were also highlighted as significant, as were a series of others, albeit less so (Table 6). In total, 20 pathways were significantly enriched with the dysregulated Model 2 metabolites, and Figure 5 shows a pathway network diagram constructed from disturbed enzymes and pathways from the Model 2 GM1T2 dataset.

**Figure 6:**
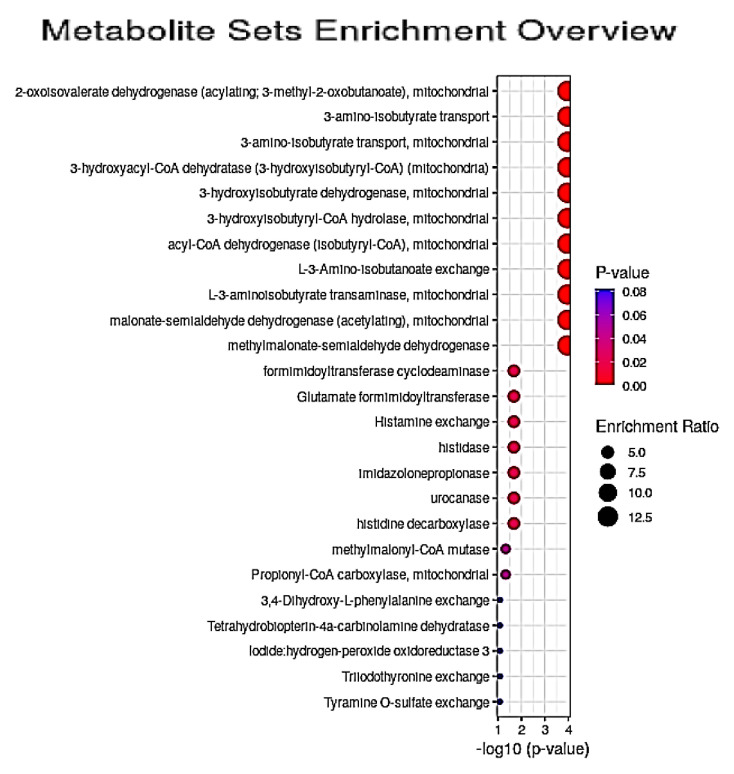
Diagram of predicted metabolite sets (PMSs) arising from a computational enzyme knockout model (the library contained a total of 912 metabolic sets that are predicted to be modified in the case of dysfunctional enzymes utilizing a genome-scale network model of human metabolism) via QMSEA with Figure 0. (dark blue) to 0.00 (intense red). All calculated *p* values indicated were corrected for multiple comparisons via the Holm–Bonferroni false discovery rate (FDR) strategy.

Secondly, PMS analysis found that 13 dysregulated enzymatic reactions and metabolite transportations/exchanges served as key features of the Model 2 dataset acquired here (Figure 6 and Table S1); significance levels for these processes ranged from < 10^−12^ to 0.012). Many of these significant pathway steps were featured in the BCAA degradation pathway, although mitochondrial 3-hydroxyacyl-CoA dehydratase (3-hydroxyisobutyryl-CoA) is involved in *n*-butyrate metabolism; mitochondrial 3-hydroxyisobutyryl-CoA hydrolase in β-alanine and propionate metabolism, as well as BCAA degradation; mitochondrial acyl-CoA dehydrogenase (isobutyryl-CoA) in the primary phase of FA metabolism; mitochondrial malonate-semialdehyde dehydrogenase (acetylating), in inositol, alanine and aspartate, β-alanine, and propionate metabolism; methylmalonate-semialdehyde dehydrogenase in inositol and propionate metabolism, in addition to BCAA catabolism; methylmalonyl-CoA mutase in the degradation of odd-chain numbered FAs, the amino acids methionine and threonine (together with valine and Ile), and cholesterol, along with the transfer of amino acid catabolites into the citric acid cycle; and finally, mitochondrial propionyl-CoA carboxylase for the carboxylation of propionyl CoA to methylmalonyl-CoA.

Finally, an analysis of metabolite sets based on the sub-cellular and cellular, organ, and tissular locations of GM1T2′s dysfunctional pathology was performed, and this revealed that fibroblasts > Golgi apparatus > mitochondria > spleen > skeletal muscle > muscle were considered to be the most significant sites for GM1T2 disease pathologies. Table 7 lists these sites, the total number of metabolites involved, the number of correct ‘hits’, FDR-corrected *p* values (ranging from < 10^−11^ to 0.033), and associated GM1T2 disease up- or downregulations in featured metabolites. Key metabolites featured include glycolysis-related glucose, and the fluid balance biomarker creatinine, the latter involved in 3 of these locations. Clearly, all these sites are of critical importance for this LSD, and their relevance, along with those for the above MPAMS and PMS results acquired, are considered further in the Discussion section below.

## 4. Discussion

### 4.1. Key Discriminatory Biomarker Variables Identified by ^1^H NMR-Linked Metabolomics Analysis

Bonferroni-corrected univariately-significant discriminatory variables identified comprised an extensive series of metabolites, which were dominated by amino acids, organic acid anions, and lipids, along with Cn and urea as waste products. With the exception of plasma total TAG concentrations, these observations were predominantly confirmed when the analysis was repeated using Ile metabolite—rather than CS-normalization (Table 3). All these potential GM1T2 biomarkers detected were extensively confirmed via the application of a series of MV and CIT analytical approaches, along with their validation requirements, e.g., Q^2^ and permutation testing for the MV PLS-DA approach. Critically, the most important key metabolite discriminators were found to be valine > lactate > Cn > total TAGs > tyrosine > glutamine > histidine > PAAR (all upregulated in GM1T2 disease, with the exception of total TAGs) from univariate analysis of CS-normalized datasets, whereas for the PLS-DA strategy, this order was lactate > PPAR > Cn > Cr/PCr > histidine > valine > formate > urea (all upregulated). Similarly, the RF CIT technique ranked the most important predictor variables in the order valine > Cr/PCr > lactate > total TAGs > leucine > Cn > glutamine (again all upregulated, except for total TAG levels).

The observation of a MV-significant variable that is not significant if subjected to one or more UV tests is a not unusual occurrence in metabolomics analyses. Possible explanations for such differences are consistency effects, specifically no univariate differences are observed because of high variance contributions of biological and/or bioanalytical sources, the escalation of false negatives (type 2 errors) arising from post-hoc ANOVA-type corrections applied to counter FDRs, and also the MV complementation (and correlation) of explanatory biomolecule variables when considered together as a whole [17,21]. These explanations are also applicable to differential ranking orders of metabolites found between UV and MV methods of data analysis.

Currently-available alternative GM1 disorder biomarkers, which are extensively reviewed in [27], include the spectrophotometric determination of aspartate transaminase (AST) in blood serum or CSF for the monitoring of CNS disease status in infantile gangliosidosis [28,29]. Moreover, epithelial-neutrophil activating peptide-78 (ENA-78), monocyte chemoattractant protein-1 (MCP-1), macrophage inflammatory protein-1α (MIP-1α), macrophage inflammatory protein-1β (MIP-1β), and tumor necrosis factor receptor 2 (TNFR2) are also employed as clinical parameters for evaluating CNS disease progression, and for therapeutic monitoring purposes, along with discrimination between the infantile and juvenile phenotypes of this condition [30].

Comparative evaluations of Ile-normalized metabolite level GMIT2:HC fold-changes with those determined from a suitable reference source which compared ^1^H NMR-determined blood serum metabolite concentrations of childhood and adulthood populations [23] confirmed that lactate, creatinine, valine, histidine, tyrosine, and phenylalanine remained all highly significantly-upregulated, low-molecular-mass biomolecules in GM1T2 plasma samples. However, although comparative evaluations could not be performed for 3-AIB, urea, the PAAR species and formate in view of the unavailability of biofluid concentration data in Ref. [23], their Ile-normalized GM1T2:HC fold-changes were all found to be highly significantly greater than a null hypothesis value of 1.00 (Table 3).

It should also be noted that such age-related fold-change comparisons involved a consideration of our blood plasma metabolite concentrations with those of blood serum in Ref. [23]. Notably, Kaluarachchi et al. [31] reported that, for standard 1D NMR analysis, 4 metabolites, specifically VLDL- and LDL-linked TAGs, lactate, and glutamine, had higher levels in serum over plasma, whereas α-glucose was higher in the latter biofluid. However, for CPMG ^1^H NMR profiles, as acquired in this study, no specific distinguishing features, such as any of those determined in our study, could be assigned. Additionally, a further study [32] reported that out of 122 metabolites investigated by non-NMR analyses, 9 of them had ‘between-biofluid’ relative concentration differences of >20%, notably arginine and specific lipid species, which were not quantified here. Although these biomolecules featured phenylalanine as one of the 3 amino acids with a significantly greater level in serum, our results will not be affected by this observation since we detected a highly significant greater GM1T2:HC Ile-normalized concentration fold-change value for it in plasma samples over that of the corresponding child:adult measure for serum in Ref. [23] (Table 3). Furthermore, such fold-changes are expected to be much less affected by this biofluid nature discrepancy than the raw metabolite levels themselves.

### 4.2. Potential Pathological Significance of GM1T2-Mediated Alterations to Plasma Metabolic Profiles Indicated by QMSEA Investigations

MSEA serves to detect biologically-relevant patterns of metabolites that are enriched significantly in quantitative metabolomics datasets [25]. This approach involves the prior selection and combination of statistically significant metabolites to determine if any informative patterns of disrupted metabolic pathways, human diseases, and the likely sub-cellular, cellular, organ, and tissue localizations for these disturbances may be discerned from predefined metabolite pathways and disease states obtained from the Human Metabolome Database [26]. The localization option is particularly valuable for the investigation of LSDs in view of the broad spectrum of irregular neurological, dysmorphic, and skeletal/organ abnormalities involved as clinical features for them (Section 4.3). The MSEA technique determines whether a dataset of functionally-related biomolecules and their concentrations are related to these pathways, diseases, and sources without any requirement to pre-select them on the basis of an established ‘cut-off’ value. It also has the ability to detect small but nevertheless consistent modifications from a group of such metabolites, and which may indeed not be detectable when using standard metabolomics practices [25,26]. Therefore, network QMSEA was conducted in the current study to explore these effects further.

#### 4.2.1. Plasma Lipoprotein-Associated Lipids and FA Metabolism

One key difference observed was the significantly lower levels of total lipoprotein-related triacylglycerols in the GM1T2 group, an observation which indicates an overall impairment in lipid metabolism in patients with this condition. However, comparisons of Ile-normalized GM1T2:HC metabolite concentration fold-changes with the corresponding child:adult values computed from Ref. [23] suggested that the difference observed may arise from age differences between the two participant groups, and therefore further investigations may be required to explore this. Moreover, since the HC cohort was fasted prior to blood sample collection, whilst the GM1T2 group was not, we would expect an increase in plasma TAG levels in the latter cohort, and hence this observation appears to be paradoxical. However, evidence for disturbed lipid metabolism has also been found in another LSD, specifically Nieman-Pick type C1 (NPC1) disorder [19]. However, in this disease, elevated concentrations of the plasma concentrations of lipoproteins and their ^1^H NMR-visible, molecularly-mobile TAG components were found in NPC1 patients, and this observation was consistent with perturbed intracellular transport, which gives rise to the build-up of cholesterol and glycosphingolipids in lysosomes and late endosomes. Similarly, in a related ^1^H NMR-based metabolomics investigation, Duarte et al. [33] found higher plasma total lipoprotein-, VLDL- and LDL-associated TAG levels in juvenile patients with glycogen storage disease type 1a (GSD1a), but lower HDL phospholipid concentrations in blood plasma when compared to an age-matched healthy control cohort (this disease is an autosomal recessive LSD in which gene expression of glucose-6-phosphatase is absent). Contrastingly, in the current study, GM1T2 disease data revealed lower total lipoprotein-associated TAGs than, and very similar HDL-phospholipid contents to, the HC group. An important feature of GM1T2 disease is the hindered intracellular transport of lipids, a process leading to an abnormal accumulation of acidic lipidic species in central and peripheral nervous system cells, particularly neurons. Hence, disease-induced modifications to the plasma levels of lipoproteins and their molecularly-mobile, CPMG ^1^H NMR-detectable TAGs, may be expected. Notably, it has been shown that a decrease in plasma HDL-cholesterol is the most common lipoprotein abnormality noted in NPC1 patients, as are diminished LDL-cholesterol and increased overall TAG concentrations [34,35]. As expected, the terminal-C**H**_3_, bulk acyl chain-(C**H**_2_)_n_-, -C**H_2_** CH_2_CO, and N-acetylglycoprotein-NHCOC**H_3_**/CH_2_-C**H_2_** -CH= mobile lipid regions of the ^1^H NMR spectral profiles noted in the current study have been previously shown to be correlated with plasma triacylglycerol levels, both in humans and mice [36,37].

Unfortunately, the highly significant downregulations in TAGs observed in GM1T2 plasma could not be considered in the QMSEA investigations conducted in view of the heterogenous, multicomponent molecular nature of individual FAs, their chain lengths and unsaturation status, their substitutional status on glycerol backbones in predominantly tri-, but also di- and monoacylglycerols (*sn*-1(3) or -2), their plasma lipoprotein densities and identities, etc. in vivo (potentially important N-acetylsugar-containing molecularly-mobile carbohydrate side-chains of ‘acute-phase’ glycoproteins were also excluded for this reason, as was the PAAR species). However, the first stage of FA metabolism, specifically the acyl-CoA dehydrogenase-catalyzed and FAD-promoted insertion of a *trans*- -CH=CH- double bond unit between the C2 and C3 sites of its acyl-CoA thioester substrate in mitochondria [38], was featured as an important altered function from the PMS analysis performed, and our results suggest that such a process may indeed be upregulated in GM1T2 patients.

Moreover, FA biosynthesis was found to be a significantly perturbed pathway in GM1T2 disease (Table 6), and this was reflected by downregulations in plasma acetate levels in patients with this condition. Therefore, this may also partially account for the diminished plasma lipoprotein-associated lipid concentrations found in the GM1T2 cohort.

#### 4.2.2. Propionate (Propanoate) and N-Butyrate (Butanoate) Metabolism

Propionate is physiologically generated as propionyl-coenzyme A from the metabolic degradation of odd carbon number FAs, and selected amino acids. Since this carboxylic acid anion has a three-carbon structure, direct entry of the propionyl-CoA product into either the β-oxidation pathway or the citric acid cycle is precluded. Subsequently, propionyl-CoA is carboxylated to D-methylmalonyl-CoA, which is then isomerized to L-methylmalonyl-CoA, the latter being catalytically rearranged to succinyl-CoA by a vitamin B_12_-dependent enzyme; succinyl-CoA is, of course, an important citric acid cycle intermediate. Significant GM1T2-mediated upregulations observed in plasma glutamate and valine were major contributors to this MPAMS analysis result.

From the PMS analysis conducted, impairments to the activity of mitochondrial 3-hydroxyacyl-CoA dehydratase (3-hydroxyisobutyryl-CoA), an enzyme involved in *n*-butyrate metabolism, was featured as the third-most significant one found (this enzyme catalyzes the dehydration of (3R)-3-hydroxybutanoyl-CoA to crotonoyl-CoA). Glutamate, which was upregulated in GM1T2 disease (Table 2 and Table 3), is a product of *n*-butyrate metabolism. Moreover, in a recent study focused on the metabolomics-based discovery of metabolic dysfunctions in mice and patients with the related GM2 Sandhoff disease [39], impairments to *n*-butyrate metabolism were also detected.

#### 4.2.3. BCAA Degradation

Highly significant GM1T2 disease-induced increases in the mean plasma concentrations of key precursors (leucine and valine), along with marginal elevations in that of the final product of valine’s BCAA degradation pathway (3-AIB), were observed in the current study, and this supplies evidence for perturbances to this important catabolic route in GM1T2 disease, as was also found in a related study targeted on pathogenic mechanisms involved in NPC1 disease [14]. Although no significant differences between mean plasma Ile levels were found in a UV context, the powerful loading of it on PC2, along with the other BCAAs, provides evidence for its MV contribution towards segregation of the HC and GM1T2 groups in PCA and PLS-DA analyses (Table 4). Consistently, PMS analysis indicated that disturbances to the mitochondrial enzymes 2-oxoisovalerate dehydrogenase (acylating; 3-methyl-2-oxobutanoate), 3-hydroxyisobutyrate dehydrogenase, 3-hydroxyisobutyryl-CoA hydrolase, L-3-aminoisobutyrate transaminase, and methylmalonate-semialdehyde dehydrogenase, together with location-dependent 3-amino-isobutyrate transport and its exchange (Figure 6 and Table S1), were further significant contributors towards the profound metabolic differences observed between GM1T2 and healthy human blood plasma samples. Notwithstanding, 3-AIB is also a terminal end-product of thymine catabolism, although as the (R) rather than the (S) stereoisomer configuration, as it is with BCAA catabolism [40]. However, both CS- and Ile-normalized plasma 3-AIB levels were found not to be significantly higher than those of the HC participants following Bonferroni-corrected univariate testing regimens.

Elevated BCAA levels in blood plasma have been observed in liver damage patients [41], and cholestatic liver disease, and hepatosplenomegaly is often a very serious or fatal complication of this condition. However, liver damage is typically absent in GM1T2 patients, which is not usually associated with organomegalies, unlike the more severe GM type 1 condition [42].

The malonate-semialdehyde dehydrogenase (acetylating) enzyme is also featured in four additional metabolic pathways, specifically β-alanine, alanine and aspartate, propanoate and inositol metabolism, although 3 of these (alanine and aspartate, propanoate metabolism) were also identified in this study as key features involved in GM1T2 chemopathology. Additionally, methylmalonate-semialdehyde dehydrogenase also plays roles in propionate and inositol metabolism. Moreover, methylmalonyl-CoA mutase is required for the deterioration of odd-chain FAs, threonine and cholesterol (the amino acid catabolites are utilized in the citric acid cycle).

However, it should also be noted that upregulated urinary 3-AIB concentrations may also arise from single nucleotide polymorphisms (SNPs) in the AGXT2 (D-3-aminoisobutyrate-pyruvate aminotransferase) gene [43], which are frequently observed in Asian populations (ca. 40% of these populations are high level excretors of this catabolite and prototypic AGXT2 substrate [44]). Hence, this paradigm may severely limit the applications of urinary 3-AIB as a diagnostic plasma or urinary biomarker for GM1T2 disease. The rs37369 SNP in AGXT2 has been found to be represent the genetic foundation of hyper-β-aminoisobutyric aciduria [43]. However, although the mean urinary 3-AIB concentrations in NPC1 patients has been found to be substantially higher than those of a corresponding heterozygous carrier group [14], ca. 20% of the population recruited to that study were of Asian descent.

Intriguingly, it has been suggested that L-leucine may exert variable effects on both catabolic and anabolic routes in vivo, and selected investigations have reported its ability to enhance O_2_ consumption and mitochondrial function [45], although an alternative study noted that this particular BCAA had the ability to promote anaerobic glycolysis, which gave rise to a diminished level of oxidative phosphorylation-dependent oxidative stress [46]. Moreover, leucine has also been found to exert favorable effects on obese, diabetic-stage rats developed in a model involving the feeding of a high-fat diet—these comprised reduced gluconeogenesis and lipid peroxidation, along with enhanced insulin sensitivity and mitochondrial function [47].

Very recently, Kaya et al. [48] reported that orally-administered N-acetyl-DL-leucine (ADLL) to NPC1 patients significantly retarded its rate of clinical disease progression, with improvements in or stabilization of a range of neurological domains. This agent also exerted beneficial effects on gait in patients afflicted with Tay-Sachs and Sandhoff diseases (GM2 gangliosidoses), and also in a mouse model of the latter. These researchers therefore concluded that the active enantiomer, N-acetyl-L-leucine, offers novel and unanticipated neuroprotective actions against these LSDs, and that the above studies support the requirement for additional assessments in future clinical trials. These observations were supported by the availabilities of extensive pre-clinical backup data.

#### 4.2.4. Ethanol Degradation

Ethanol metabolism was also noted as a highly significant catabolic pathway in view of the decreases in plasma acetate level noted in GM1T2 patients, although this was not found to be statistically significant in a univariate context. This hepatic pathway involves the conversion of the ethanol to acetaldehyde, and then to acetate and acetyl-CoA, which serves as a citric acid cycle substrate. Such downregulations in plasma acetate levels may therefore reflect an imbalance in one or more of the enzymes involved in these reactions, although further investigations will be required to substantiate this. Intriguingly, excessive ethanol exposure is known to perturb lysosomal protein degradation [49,50], and subjection of rats to chronic ethanol administration powerfully interferes with lysosome biogenesis as well as lysosomal proteolysis [51,52]. Indeed, one report [53] found that hepatic levels of autophagic vacuoles (AVs) were increased in a mice model which involved the acute administration of ethanol to these animals, and that this effect was dependent on the metabolic oxidation of ethanol. These investigators suggested that reactive oxygen species (ROS) are generated from this oxidation, and that these oxidants suppress the activity of the mechanistic target of the rapamycin complex 1 (MTORC1, a complex with serine/threonine protein kinase activity of molecular mass 250 kDa); such ROS now represent key features of the pathological mechanisms involved in LSDs, e.g., [39]. However, acetaldehyde is itself highly reactive towards the free amino and thiol functions of selected amino acid residues in proteins usually via Maillard or Michael addition reactions [54] (e.g., lysyl/N-terminal and cysteinyl residues respectively, although primary amino functional groups also participate in Michael addition reactions). Therefore, it is conceivable that this aldehyde directly inhibits MTORC1 via its reactivity with selected amino acid residues therein.

Therefore, lower levels of acetate observed in the blood plasma of GM1T2 patients may reflect their relative inability to oxidize endogenous toxic acetaldehyde to this analyte, and/or any endogenous ethanol available to acetaldehyde, and these observations may be of both pathological and diagnostic significance. To date, there are no reports available regarding the dysregulation of alcohol and aldehyde dehydrogenases in LSDs.

However, ethanol, which is readily ^1^H NMR-detectable in human biofluids such as blood plasma and urine, was undetectable in all plasma samples explored in this study, including those from the HC group; the information sheet for participants instructed them not to consume alcohol within the 24 h period prior to sample collection.

#### 4.2.5. Seleno-Amino Acid and Glutathione (GSH) Metabolism

Perturbations to seleno-amino acid metabolism, which were indicated by MPAMS analysis as being highly statistically significant following FDR correction (Table 4), were supported by the observation of strong downregulations in plasma alanine levels. Selenocysteine is an essential proteinogenic amino acid for at least several enzymes, including glutathione peroxidases, thioredoxin reductases, methionine-R-sulfoxide reductase B1 and formate dehydrogenases [55]. Of course, glutathione peroxidase is essential for combating oxidative stress in cells through the direct neutralization of H_2_O_2_ to water and O_2_, in addition to the conversion of alkyl peroxides in general to alcohols and water [56]. H_2_O_2_ acts as critical precursor of the aggressively-reactive and DNA-damaging hydroxyl radical (^●^OH) [57]. Thioredoxins are involved in intracellular redox-signaling, and effectively respond to oxidative stress in a protective function; indeed, they reduce oxidized cysteinyl (cystinyl) residues via cleavage of their disulfide bonds [58]. A further selenium-containing amino acid is selenomethionine [55]. Since mitochondria represent an important source for the deleterious generation of excessive levels of ROS, further details regarding their importance in this context is discussed below in Section 4.3.3.

Defects in GSH metabolism were implicated as an important metabolic set imbalance from the upregulations in plasma glutamate CPMG spectrum ISB resonance intensities observed in the GM1T2 patient cohort (mean fold-change 1.14 for CS-normalized ISB intensities). Since the reduced form of this tripeptide is a powerfully defensive endogenous antioxidant [59], and is also the substrate for glutathione peroxidase, disturbances in its metabolism may serve as an important feature of GM1T2 pathogenesis. Indeed, it is present at high millimolar levels (ca. 5 mmol./L) in many human cell types. Imbalances in ROS generation, notably that from the mitochondrial respiratory chain, have been speculated to represent key features of both NPC1 [60] and GM2 (Sandhoff) [39] diseases, and their excessive production in these conditions may serve to generate ‘foreign’ ROS-induced oxidation products, which have the capacity to trigger inflammatory cascades via chemotaxis. Indeed, central nervous system (CNS) inflammation is a characteristic of pathogenesis in mouse models of GM1 and GM2 gangliosidoses [61].

A further consideration is the occurrence of one form of glutathione peroxidase as a non-selenium-dependent one, and Lawrence and Burk [62] found that the proportion of the activity of this form in the liver varied from 35% in rats to 100% in guinea pigs. They also found that non-selenium-dependent glutathione peroxidase activity was limited to the ‘soluble’ compartment of rat liver.

#### 4.2.6. Aspartate Metabolism

Importantly, imbalances in the mitochondrial metabolism of neuroactive aspartate represents an important source of brain biomarkers for Alzheimer’s disease, a further neurodegenerative condition [63]. Transport of acetyl-CoA into mitochondria occurs via two major routes following its transformation to either citrate or N-acetylaspartate. The first of these involves the reconversion of mitochondrial citrate to acetyl-CoA via ATP-citrate lyases, whereas the second features the liberation of mitochondrial acetate from N-acetylaspartate, which is then reconverted back to acetyl-CoA via the actions of acetyl-CoA synthetase. For these shuttle processes, the present study found that both citrate and glutamate were upregulated in GM1T2 blood plasma for the first route, whereas glutamate alone was upregulated for the second. Since key plasma metabolites from both these mitochondrial shuttles are significantly modified in GM1T2 disease, this observation may indicate a more epitomized mitochondrial dysfunction in general, which is discussed in Section 4.3.3 below.

#### 4.2.7. Amino-Sugar Metabolism

Distinct GM1T2 disease-mediated changes in the plasma concentrations of neuroactive glutamate and glutamine (upregulations), and acetate (downregulation) provides powerful support for disturbed amino-sugar metabolism in this condition, and this observation is fully consistent with known mechanisms for the chemopathology of this LSD. Since GM1 conditions arise from deficiencies of β-galactosidase with respect to keratin sulfate, asialofetuin, oligosaccharides equipped with terminal β-linked galactose residues and lactosylceramide, along with gangliosides themselves [64], the accumulation of GM1 gangliosides in visceral organs, especially the central nervous system, is a hallmark of GM1 gangliosidosis.

Physiologically-active gangliosides comprise an N-acetylneuraminate head group covalently attached to a ceramide molecule which principally retains them on the cell membrane’s outer leaflet. Their multiple biological properties include roles as cell surface receptors and markers, and intracellular communication agents; they also take part in cellular cycling and mobility, and the fine-tuning of cellular signaling [65,66].

One major bioanalytical consideration is that the acetamido-CH_3_ function singlet signals of N-acetylsugars, which in human plasma predominantly comprise those of terminal-N-acetylneuraminate and bulk-chain N-acetylglucosamine residues of the carbohydrate side-chains of ‘acute-phase’ glycoproteins (δ = 2.03 and 2.07 ppm) [67], were not markedly elevated in GM1T2 disorder samples, as might be expected in view of its inflammatory nature; indeed, CNS inflammation serves as an important characteristic of the pathogenesis of both GM1 and GM2 gangliosidoses [30,61]. However, this observation is not directly comparable to the findings made in [68], since the low-molecular-mass N-acetylated saccharide derivatives monitored were detected in the urinary profiles of these patients.

As might be expected, elevations in low-molecular-mass N-acetylsugar concentrations such as that of N-acetylneuraminate in biofluids predominantly serve as the basis for dysfunctions in the lysosome [25], as indeed they do from an examination of the urinary metabolic profiles of NPC1 patients [14]. However, it was not possible to determine these in our ^1^H NMR plasma profiles since firstly, any sharp singlet resonances arising from such low-molecular-mass N-acetylsugar-containing saccharide species will be largely overlapped by the much higher intensity and broader ‘acute-phase’ glycoprotein signals, and these interferants are themselves overlapped by the similarly broad lipoprotein-associated TAG-CH_2_-CH=CH- function signal, as indeed they were in an NMR-based metabolomics study focused on evaluations of the metabolic profiles of NPC1 disease patients [20]. Secondly, such species are likely to remain bound to plasma proteins such as albumin and gamma-globulins, and hence be ‘NMR-invisible’, and concentrations therein may be too low for ^1^H NMR detection in any case, even at an operating frequency as high as 700 MHz as employed here.

However, N-acetyl function acetamido resonances arising from N-acetylamino acids may also be employed as urinary biomarkers in patients with selected inborn errors of metabolism, Indeed, in 2003 Krawczyk and Gradowska [69] developed an ^1^H NMR method for the analysis of urinary N-acetylaspartylglutamate in Canavan disease.

#### 4.2.8. Alanine Metabolism

The down- and upregulations in alanine and glutamate, respectively, observed in the current study point to dysregulations in alanine metabolism, which was another significant pathway found in the HPAMS analysis performed (FDR-corrected *p* value 4.25 × 10^−5^). Indeed, these metabolites are also key components of the larger glutamate, alanine, and aspartate metabolic pathway, and aspartate metabolism per se was also featured as a very highly significant one from this analysis conducted (Section 4.2.7). Alanine is biosynthesized from either pyruvate or BCAA sources: the former route involves a two-stage reductive deamination process, the first of which reacts 2-oxoglutarate with ammonia to glutamate and H_2_O via glutamate dehydrogenase_s_ and is NADH-promoted, the second featuring transfer of the glutamate product’s amino function to pyruvate to form alanine and a regenerated 2-oxoglutarate species (via an aminotransferase enzyme) [70]. The reverse oxidative deamination reaction is catalyzed by the same enzymes, its direction being predominantly dependent on the relative levels of substrates and products available. Acetate is also featured in this process, and hence its downregulation, albeit a minor one, observed in GM2T1 disease plasma samples also indicates its involvement in this pathway.

Since all cells are rich sources of pyruvate, the above transamination reactions are readily reversible, and alanine is strongly associated with other metabolic pathways, e.g., glycolysis, gluconeogenesis, and the citric acid cycle [70].

Moreover, Broer et al. considered alanine metabolism, and its transport and cycling in the brain [71], and provided evidence that it acts as a critical ammonia transfer carrier (cycling of brain glutamate and glutamine remains incomplete in the absence of ammonia return to glial cells). For this purpose, these researchers explored the transport and metabolism of alanine in guinea pig brain cortical tissue slices and prisms, in neuron and astrocyte primary cultures, and in synaptosomes. They found that although its uptake by neurons was mainly moderated by Na^+^-dependent transporters with the system B(0) isoform of the sodium-dependent neutral amino acid transporter (B(0)AT2)-mimetic actions, that by astrocytes was controlled by system L isoform of the linker for activation of T-cells family member 2 (LAT2). Notwithstanding, under their experimental conditions, alanine did not supply any significant levels of carbon sources for energy, nor neurotransmitter metabolism. Therefore, GM1T2 disease-mediated reductions in available plasma alanine concentrations, along with upregulations in those of glutamate and glutamine, may exert a significant influence on this process.

### 4.3. QMSEA of GM1T2 Pathological Localisations Determined from Metabolic Disturbances Observed

#### 4.3.1. Fibrobasts

Fibroblasts represent important cell types in GM1 diseases, not least because of their important involvement in the development of enzyme replacement therapies (ERTs). Indeed, fibroblasts, in addition to white blood cells, have a deficient level of β-gal enzyme activity in both GM1 gangliosidosis and galactosialidosis disorders. Interestingly, Condori et al. [72] successfully performed an ERT study involving genetic fusions of the plant galactose/galactosamine-binding lectin, (the B-subunit of ricin, abbreviated RTB), and the human acid β-galactosidase (β-gal) enzyme, employing a biogeneration system based on plants (both the β-gal:RTB and RTB:β-gal fusion adducts retained lectin and β-gal activities). They found an efficient take-up of both fusion orientations of the purified proteins into GM1 patient fibroblasts, and this process gave rise to an alleviation of the GM1 ganglioside substrate level with activities similar to those of emanating from mammalian cell β-gal.

Hence, fibroblasts represent cornerstones for the biosynthesis of GM1, and exogenous GM1 protects against apoptosis via its ability to promote the synthesis of sphingosine-1-phosphate [73]. Although evidence for major functional activities of gangliosides in fibroblasts has been obtained, such behavior is dependent on their specific molecular nature, and the particular organ sources of these cells. Indeed, fibroblasts from different organs, e.g., those arising from skin and oral tissues, have demonstrated differential contents of the glycosaminoglycan hyaluronate, and differential growth responses following transforming growth factor-β1 (TGF-β1) cytokine stimulation [74].

Moreover, a permanent human cell line which preserved the defect of the lysosomal enzyme G_M1_-1019-SV was developed from fibroblasts collected from GM1 patients via transfection conducted with replication origin-minus simian virus 40 DNA [75]. During >120 population doublings, such cell lines grew rapidly without undergoing senescence. Notably, β-gal activity in these cells was 40-fold lower than that of normal fibroblasts.

#### 4.3.2. Golgi Apparatus

GM1 represents a critical constituent of membrane microdomains in many cell types, and is biosynthesized in the Golgi apparatus by a specific glycosyltransferase; cell surface levels of GM1 are dependent on the expression of this enzyme [76]. These microdomains are located at the leading edge in polarized cells, the polarization process involving a series of co-ordinated cellular rearrangements that prepare cells for migration. The Golgi apparatus is also oriented towards this leading edge on polarization, and it has been hypothesized that this contributes towards plasma membrane asymmetry. Since the mechanism featuring the asymmetric accumulation of GM1 was unclear, Bisel et al. (2013) [77] explored the decoupling polarization of GM1 and the Golgi apparatus within the plasma membrane, and found that the regulation and reinforcement of directional selection in cell migration processes may occur via a synergistic, albeit independent biochemical mediation of Golgi apparatus polarity by (1) methylerythritol 4-phosphate/extracellular signal-regulated kinase (MEP/ERK), and (2) phosphoinositide 3-kinase (P13K).

The biosynthesis of gangliosides GM1 and GM2 in intact rat liver Golgi vesicles is stimulated by phosphatidylglycerol as much as or even more so than by detergents (Triton X-100 and octyglucoside, respectively) [78].

#### 4.3.3. Mitochondria

In LSDs, both lysosomal dysfunction and diminishing autophagic fluxes exert a major impact on mitochondrial function, and these flux perturbations may occur during the final stage of the autophagy process, i.e., autophagosome clearance, in which the lysosome has an essential involvement. Indeed, the autophagic clearance of malfunctional mitochondria serves as an important criterion for their quality control [79]. A significant number of damaged mitochondria and autophagosomes remain in the cytosol, an observation indicating a perturbed and incomplete autophagic flux [80].

In GM1 gangliosidosis mouse models, GM1-ganglioside accumulates in the glycosphingolipid-enriched microdomain (GEM) components of mitochondria-associated endoplasmic reticulum (ER) membranes (MAMs) in the brains of these animals [79]. In this location, it has been suggested that this ganglioside interacts with the phosphorylated class of inositol triphosphate receptor-1, a process which influences this channel’s activity, and inducing a calcium ion (Ca^2+^)-mediated-ER stress response. Subsequently, Ca^2+^ ions passage into mitochondria, a process resulting in a localized overload with this metal ion, along with activation of the mitochondrial apoptotic pathway [81]. β-Galactosidase (−/−) mouse astrocyte mitochondria were found to be morphologically abnormal, with a diminished mitochondrial membrane potential (ΔΨm) [82].

One major route for the adverse generation of intracellular ROS is mitochondrial energy metabolism [78]. Such ROS, including superoxide anion and hydrogen peroxide (O_2_^●-^ and H_2_O_2_ respectively), which may both serve as important precursors to the aggressively-reactive hydroxyl radical (^●^OH), which reacts at diffusion-controlled rates with many critical biomolecules such as DNA, proteins, and polyunsaturated FAs (PUFAs); in the absence of sufficient protective batteries of intracellular antioxidants, these adverse reactions may cause much damage to cells and tissues [83]. Currently, much evidence is available that enhanced levels of oxidative stress induced by ROS are involved in the chemopathologies of neurodegenerative disorders, including Alzheimer’s, Huntington’s, and Parkinson’s diseases [84]. Moreover, inflammatory responses featured in Sandhoff disease also lead to enhanced levels of oxidative stress (OS), which may give rise to neural damage and death [39]. Intriguingly, Vasquez et al. [60] suggested that OS represents a key mechanistic feature involved in the etiology and pathogenesis of NPC1 disease. Moreover, autophagy activation coupled with an enhanced sensitivity to OS can give rise to mitochondrial dysfunction [82,85].

The dysfunctions in seleno-amino acid and GSH metabolism found in this study (Section 4.2.5) suggest that intracellular antioxidant responses to excess ROS production, i.e., those of glutathione peroxidase and its GSH substrate, may be impaired in GM1T2 disease. However, for an investigation involving astrocytes, GSH and GSH-associated enzymes (glutathione reductase (GR), glutathione peroxidase (GPX), and glutathione-S-transferase (GST)) were found to be of high abundance in their cytosolic fractions, whereas those present in their mitochondrial pools were relatively low [86]. Nevertheless, since they remain important sites of both O_2_^−^ and H_2_O_2_, GSH-, and GSH enzyme-containing neural cell mitochondria may serve to offer significant barriers against the biomolecular attack of toxic and chemically-reactive ROS within the nervous system. Notably, differences observed between the sub-cellular distributions of these species in differing neural cells may serve as a therapeutically-relevant platform for the distinctive expression of neurotoxicity in selected cellular and/or sub-cellular compartments.

As noted in [39] for Sandhoff disease, the biosynthesis of GSH is linked to oxidative stress and inflammatory cascades, and disruptions in energy supply may give rise to an enhanced mitochondrial respiratory chain activity in mitochondria, a process engendering the deleterious generation of ROS, including the aggressively-reactive ^●^OH radical if conditions for its formation are optimal. Hence, activation of GSH pathways such as its biosynthetic one implicated in the current study may be required to combat and scavenge toxic ROS species. Consistently, it is now clearly accepted that lysosomes play important roles in the maintenance and efficient healthy functional status of other intracellular organelles, especially mitochondria [87].

Both autophagy and protein catabolism may be activated by enhanced requirements for energy, as was found in an investigation focused on MPS I and VII mice [13]. Moreover, Ou et al. [39] found increased brain concentrations of amino acids, along with their derivatives and dipeptides, in Sandhoff disease mice, and this observation is consistent with upregulated protein catabolism. In this study, we found upregulations in the plasma levels of no fewer than 10 amino acids, of which 7 were statistically significant, and this may also reflect an increased level of protein catabolism. Such energy deficits can also trigger lipid and carbohydrate metabolism, induced by a diminished adiposity, which is indeed frequently encountered in subjects with LSDs [88]. Similarly, an enhanced level of membrane lipid biosynthesis, which is activated by the inflated lysosome and associated cellular swelling, will also be expected to exert an effect on lipid metabolism, and our observation of markedly decreased plasma lipoprotein-associated TAG levels, and dysregulations in associated FA biosynthetic routes (Table 4), support this. Indeed, depletions in essential precursors arising from lysosomal actions may exert a critical influence on the extent of autophagy, in addition to the general metabolic status of patients afflicted with GM1T2 disease.

Interestingly, in a proteomic analysis of brain in a mouse model of mucopolysaccharidosis I, Ou et al. [89] discovered a cytoskeletal system abnormality, which was partially ascribed to a modified cellular architecture arising from adverse storage accumulation.

The determination of CSF lactate levels has been previously proposed for investigations of electron transport chain inborn errors. Fortunately, Hutcheson et al. [90] explored reference concentration ranges for this CSF marker in children, and also its relationship with its corresponding plasma lactate levels (median values were 1.4 and 1.5 mmol./L respectively). CSF lactate was found to be ≥3.0 mmol./L in 8/11 children with mitochondrial electron transport chain disorders; however, 2 of these also had normal plasma values, and therefore in principal CSF lactate can be upregulated despite this marker being within the normal level range for plasma. Hence, if CSF lactate is raised significantly in GM1T2 patients in view of mitochondrial abnormalities, then plasma lactate values may also be elevated, as indeed they are in the current study. Notably, in [89], the correlation observed between CSF and plasma lactate levels was not very strong (R^2^ = 0.14). Moreover, hypoxia, rapid exertion (e.g., as in a seizure), sepsis, cardiac disorders, and chronic diseases in general, may also give rise to significant increases in plasma lactate [91]. Although commonly utilized as a metabolic abnormality measurement, including lactic acidosis, this agent also exerts toxic actions towards neurons when present at high concentrations [92]; notably, the primary stage of cerebral ischemia involves the accumulation of lactic acid, and consequently the brain’s pH value is lowered to values within the 6.0–6.7 range.

#### 4.3.4. Spleen and Muscular/Skeletomuscular Systems

Although only marginally significant, the spleen, and muscular/skeletomuscular systems were organ and tissue localizations also displaying statistical significance in the QMSEA modeling experiments conducted. Such observations are highly relevant to the GM1 disease process, since children with the GM1 type 1 disorder develop an enlarged spleen and liver (hepatosplenomegaly), and skeletal abnormalities, together with seizures, marked intellectual disabilities, joint stiffness, distended abdomen, muscle weakness, gait problems, and corneal clouding [1,2,3]. However, for the GM1T2 condition, only mild skeletal abnormalities are observed, along with a slowly progressive, more generalized neurodegeneration (with an onset ranging from 7 months and 3 years of age) [1]. Furthermore, usually GM1T2 disease is not associated with organomegaly and corneal clouding problems.

Notwithstanding, one study based in Brazil [93] reported on 12 subjects from 10 unrelated families with type 2 and type 3 GM1 disorder (*n* = 4 and 8, respectively), and found that at onset, 6 of these presented with skeletal deformities, the remaining 6 with neurological symptoms. Moreover, 9/12 of them had a noticeable muscle atrophy.

#### 4.3.5. Consideration of Marginally Significant QMSEA Location-Based Metabolite Sets

Marginally significant contributions from the bladder, pancreas, and prostate gland were also notable in this study. However, to date, there appears to be only very limited literature available on urinary sequelae in LSDs. Gografe et al. [94] demonstrated a mononuclear cell infiltrate located in the lower urinary tract in a mouse model of mucopolysaccharidosis type IIIB, and this gave rise to urinary retention, which has not, however, been confirmed in humans. Notwithstanding, one case report of a patient with Hunter syndrome revealed a neurogenic bladder disorder, although this was a secondary development to cervical myelopathy [95]. Furthermore, in 2014 McNamara et al. reported, for the first time, a neurogenic bladder dysfunction in a neuronopathic Gaucher disease patient [96].

Literature information available on associations of prostate gland conditions with LSDs is very sparse. However, one study [97], which explored linkages between Gaucher disease and cancer incidence, found that cancer sub-groups, including prostate cancer, failed to provide any evidence for statistically significant higher risks. Similarly, there is little or no information available on pancreatic function in LSDs, although it has been found that the induction of autophagy in pancreatic ductal adenocarcinoma arises as part of a wider transcriptional process that controls the activation of lysosome biogenesis and function, along with nutrient scavenging, which is mediated by transcription factors [98].

Notwithstanding, Gray-Edwards et al. [99] recently reported that from a clinical viewpoint, modifications in blood biomarkers monitored by them (specifically aspartate aminotransferase, lactate dehydrogenase, plus Ca^2+^, Cn, and albumin levels) may provide indications of muscle atrophy, hepatosplenomegaly, and/or progressive cachexia, which all represent recognized sequelae of GM1 gangliosidosis disorder, and also further LSDs (this study is further reviewed in Section 4.4 below).

### 4.4. Overview of Potential Contributions of Dysregulated Plasma Biomolecule Concentrations, Metabolic Pathways and Pinpointed Disorder Locations to GM1T2 Disease Pathogenesis

To date, information available regarding the impact of metabolic deficiencies and associated physiological alterations on GM1T2 disease progression remains limited. The major purpose of the lysosome is the recycling of biomolecular macromolecules to their constituent monomers for biological harnessing, and one or more deficiencies of key lysosomal enzymes can give rise to the deleterious build-up of undegraded or partially-degraded high-molecular-mass substrates, along with a stimulation of primary biosynthetic routes for these substrates in view of deactivated recycling processes. Therefore, demands for energy and energy sources by these cells would be expected to escalate in view of an increased biosynthesis of gangliosides, and then lead to an enhanced mitochondrial activity which, in turn, will produce an excessive level of chemically-reactive ROS, which can exert damaging effects towards many critical biomolecules and cells. Our data are therefore consistent with those of Ou et al. [39], who offered a similar explanation for results acquired in a study focused on metabolic profiling in Sandhoff disease, in which increased ROS generation was suggested to give rise to cell damage and hence inflammatory processes, which are major features of disease progression in GM2 gangliosidoses [61].

One recent study explored novel biomarkers for human GM1 gangliosidosis disease, and also their ability to effectively respond to the clinical efficacy of GT in a feline model system [98]. For this purpose, a panel of such biomarkers were developed using blood, CSF, urine, 7 T magnetic resonance imaging (MRI), and coupled single-voxel in vivo ^1^H magnetic resonance spectroscopy (MRS) in GM1 cats, and these parameters were compared to those acquired on untreated human GM1 patients. Of the many biomarkers determined, the most promising were found to be N-acetylaspartate, and in view of a significant spectral overlap, an MRS combination of glycerophosphocholine and phosphocholine (GPC/PCh), which were GM1-upregulated biomolecules monitored directly in the brain, and which normalized following GT in cats. Although it was not possible to detect and determine N-acetylamino acids in our plasma ^1^H NMR profiles in view of major overlap from highly intense N-acetylated glycoprotein and lipid resonances within the acetamido proton region (δ = 1.95–2.20 ppm), HDL-associated phospholipid concentrations were found not to be significantly different from those of the HC group. Moreover, in [99], CSF concentrations of lactate dehydrogenase (LDH) and aspartate aminotransferase (AST), which are known to be increased in patients with other LSD and CNS conditions [100,101,102], were found to be related to the CNS disease severities of both GT-treated and untreated GM1 cats. Disturbances in the blood-brain barrier may allow plasma LDH to reach the CSF, although its generation by neoplastic tissue, or by white blood cells and exogenous bacterial sources, may also be responsible for this observation [103,104,105].

Further MRS-detectable metabolic modifications found in [99] were in the cerebellum, the most altered voxel observed in untreated feline GM1 disease, where, with the exception of the glutamate neurotransmitter and its glutamine precursor, all biomolecules monitored had significantly different contents than those of the healthy group. These comprised the neuronal markers N-acetylaspartate alone (↓ at 4 and 8 months ages) or combined as overlapping MRS signals with and N-acetylglutamate (↓ at 8 months only), the glial cell biomarker *myo*-inositol (↑ at 8 months), the demyelination markers GPC/PCh (↑ at 4 and 8 months), and an MRS signal composite of the metabolism markers Cr and PCr (↑ at 8 months only); ↑ and ↓ symbols correspond to up- and downregulations, respectively. In cats receiving GT, all GM1-altered metabolite levels were ameliorated either partially or completely at a time-point of >3 years post-injection.

Metabolite concentrations from the cerebellar voxel were found to be strongly related to clinical signs. Indeed, N-acetylaspartate was strongly and positively correlated with clinical function, and higher levels of this biomarker correctly predicted improvements in clinical status. However, GPC/PCh and inositol phosphate were found to be negatively correlated to neurologic function.

However, glutamate and glutamine were significantly upregulated in the corona radiata voxel of GM1 disease cats at an age of 4 months only. These values were normalized following GT, as they were in the thalamus [99]. Hence, elevated plasma levels of these amino acids found in GM1T2 plasma may be related to these observations.

Since Cr may be directly converted to Cn via a non-enzymatic dehydration process, or indirectly via a PCr intermediate generated from the actions of Cr kinase [106], the GM1T2-upregulated concentrations of plasma Cn observed in the current study may be associated with significantly higher cerebellar GM1 Cr and PCr contents. A plot of the raw intensity of the plasma Cn resonance to that of Cr/PCr was linear and highly significant (r = 0.85, *p* =2.30 × 10^−8^), and this provided evidence for the same or related metabolic sources for these metabolites (the y-intercept of this plot was also significantly greater than zero, *p* = 0.017). However, no significant difference in the [Cr/PCr]:[Cn] signal intensity ratio was found between the HC and GM1T2 classifications. Although Cr is a highly significant contributor to the Cr/PCr signal, it is also likely to contain contributions from PCr in view of very similar chemical shift values for these biomolecules’ -CH_2_ function resonances (δ = 3.93 (this study) and 3.936 [21], respectively at pH 7.0). Although there is only a sparse level of scientific literature reports available on the presence and detectability of PCr in human plasma, it has been found to be present in extracts of whole blood [107]. Furthermore, Griffiths [108] reported that only traces of PCr and Cr phosphokinase were found in a mixed population of young erythrocytes, but not in mature cells, and therefore it appears that a significant proportion of whole blood PCr is present within the extracellular plasma matrix, although its ^1^H NMR distinction from Cr remains problematic.

As reported in [99], blood markers monitored in humans, i.e., in infantile, late infantile, and juvenile GM1 patients, revealed that although hypocalcemia was detected in the youngest age group, this was not the case for the late infantile and juvenile (GM1T2) ones. The extent of hypoalbuminemia in these patients was found to be enhanced with disease severity, and varied from no significant decreases observed in juvenile patients to pronounced ones in infantile ones; this parameter was also noted in GM1 cats irrespective of disease time-point, and was improved following GT treatment. However, blood Cn concentrations were found to be diminished somewhat in late-infantile and juvenile patients when expressed relative to those of the infantile cohort. Although it appears that the latter disease classification had significantly lower mean blood Cn values than that of age-matched HCs, individual Cn level fold-changes for the other two GM1 groups ranged from 0.8–1.2 and as much as 0.5–1.5 for the infantile and juvenile patients, respectively. Therefore, the higher values observed for the latter group are more consistent with our findings of a mean fold-change value of 2.65 found here for this marker in GM1T2 plasma samples; however, the ‘between-participant’ coefficient of variation of CS-normalized plasma Cn levels in this group was only 11%. In addition to the availability of higher levels of Cr and PCr precursors for Cn synthesis/biosynthesis, possible explanations for this ^1^H NMR-detectable upregulation are provided in Section 5.

Finally, if and when ERT is established as a valuable therapeutic strategy for GM1T2 disease, in principle the biomarkers discovered here may be validated against this form of treatment in both humans and animal models.

### 4.5. Comparative Evaluations with Metabolomics Datasets Acquired on Plasma/Serum Samples Collected from Patients with other LSDs

Unfortunately, previously conducted untargeted NMR-linked metabolomics investigations of LSDs, which are focused on blood plasma or serum as a bioanalytical matrix, are limited to only two conditions, namely NPC1 [20] and GSD1a [33] diseases. In conjunction with upregulated VLDL-TAG terminal-CH_3_, HDL/LDL/VLDL bulk chain-(CH_2_)_n_- and further altered lipid resonances, univariately-significant NPC1 disease-mediated enhancements of plasma concentrations of Ile, valine, alanine, arginine, proline, glutamate, histidine, and phenylalanine, and also that of butane-1,2-diol, were reported in [20]. As observed here, this NPC1 study discovered disturbances in the metabolism of a wide array of both essential and non-essential amino acids, and with the exception of proline and arginine, all of these were featured as GM1T2 biomarkers in the current study (again all upregulated, excluding alanine).

In addition to disturbances in lipoprotein TAG profiles discussed in Section 4.2.1, GSD1a disease blood plasma samples collected from juveniles was found to have statistically-significant upregulations in lactate, acetate, the ketone bodies acetone, and 3-D-hydroxybutyrate concentrations, and in Cr:Cn concentration ratios [33]. Furthermore, downregulated plasma glucose was also observed in patients with this condition, and a tentatively-assigned ^1^H NMR α-hydroxy-butyrate biomarker signal for GSD1a disease was also found. Moreover, this study reported plasma Cr:Cn ratios of 0.9 and 3.6 for age-matched HC and GSD1a participants, respectively. However, since we found an at least partial superimposition of PCr and Cr resonances at ca.3.95 ppm and an operating frequency of 700 MHz, we computed (Cr + PCr):Cn ratios for our study’s HC and GM1T2 cohorts, which were only 0.61 ± 0.22 and 0.68 ± 0.09 (mean±SEM) respectively (*p* >0.05, ANOVA). As tentative comparators, these ratio indices are more similar to those of the HC group of the GSD1a study than they are to the LSD patients evaluated [33].

Hence, it appears that the blood-plasma-based biomarkers identified here may be transferable for application to the diagnosis and prognostic monitoring of other LSDs. Additionally, future ^1^H NMR-linked MV comparisons of contributory biomarkers may indeed serve as valuable strategies for exploring the similarities or distinctiveness of perturbations to metabolic patterns of differential LSDS. Assuming similar collection methods and sampling collection tubes used, or after making sufficient allowances for any such differences in these approaches, future studies focused on the meta-analysis of untargeted metabolomics datasets from a range of LSDs or related disorders may be reliably conducted.

Recently, an extensive review of literature reports available on metabolomics- and biomarker-based investigations of lipid storage diseases was made available [21]. This review confirmed the sparsity of literature reports focused on untargeted blood plasma/serum-based metabolomics investigations of such LSDs, although an increasing number of these are now available with the employment of urine as a tracking medium, e.g., [14]. However, the prevalence of those focused on more targeted blood-based investigations are now steadily increasing, e.g., cholesterol oxidation products/7-ketocholesterol for NPC1 disease, and sphingolipids for Fabry disease, etc. [21].

## 5. Potential Limitations of the Study

One major limitation of this study is the absence of one or more suitable control groups of blood plasma samples collected from patients afflicted with other, non-GM1T2 LSDs in order to confirm that our metabolomics findings are sufficiently specific for the diagnosis of this debilitating condition alone, and not others. However, in view of problems associated with the availability and accessibility of samples from such very rare LSD cases, the investigators elected to make comparisons of the results acquired in this study with those arising from the only two previously reported ^1^H NMR-based metabolomics studies of blood plasma collected from patients with NPC1 and GSD1a diseases (Section 4.5).

Evidence provided suggested that one of the most significant predictor variables found in this metabolomics study was total TAG levels (mainly vLDL- and LDL-associated lipids); however, one limitation of this is that these significant macromolecules, which were downregulated in GM1T2 disease plasma samples, may arise from the differential dietary regimens associated with age differences between GM1T2 and the control participants, i.e., the GM1T2 cohort was somewhat younger (Section 2.2). However, application of P-RDA permutation tests found that neither the age, nor gender predictor variables provided any significant contributions to any ^1^H NMR ISB variances, but disease status did. A further major limitation of this study was the fasting process which our protocol instituted for the HC participants, but not so for the GM1T2 patient cohort; usually, chylomicrons disappear from the human circulation following overnight fasting episodes [109]. Nevertheless, this problem was not readily avoidable since it is very difficult to achieve the execution of a satisfactory pre-biofluid sampling fasting program in GM1T2 patients in view of the debilitating nature of their disease course, together with the intensive and strict nature of their attentive clinical management. However, conversely it was found that total plasma lipoprotein-associated TAG levels were significantly higher in the pre-fasted HC group than they were in GM1T2D patients. Moreover, although we may expect plasma Cn levels to significantly rise with increasing participant age [110], the converse effect was observed with the younger GM1T2 patient cohort (plasma and serum Cn concentrations are usually routinely used to monitor renal function in the management of selected clinical conditions [104]). Interestingly, Cn production may also be diminished in patients with low skeletal muscle mass, and hence serum/plasma concentrations of it have been employed as a surrogate measure of muscle mass [111]. Although muscle atrophy is a relatively common symptom in GM1 type 1 and GM1T2 diseases, our observation of upregulated Cn in GM1T2 patients is at variance with the expected decrease, and therefore this observation requires an alternative explanation. Indeed, Cn is featured as a potential biomarker associated with muscle, skeletal muscle, and spleen localizations of dysregulated metabolism and metabolic pathways in this LSD (Table 5); nevertheless, for muscle and skeletal muscle, it would be expected to be downregulated in associated biofluids and tissues.

Notwithstanding, plasma Cn levels may also be significantly affected by both glomerular filtration rate (GFR)- and non-GFR-linked variables, including dietary, exercise, pregnancy, and stress parameters, along with kidney disease and age [111]. Furthermore, the GM1T2-induced upregulations of it found here may also be ascribable to dietary sources and the differential fasting status of the two groups of participants involved in this investigation. Indeed, creatine in dietary meat is chemically transformed to Cn during high temperature cooking processes [112], and is readily absorbed following ingestion, a process conceivably reinforcing and enhancing plasma Cn levels. Interestingly, Herder et al. [113] discovered that springbok antelopes with a GM2 gangliosidosis disorder resembling human Sandhoff disease also presented with polycystic kidney disease—human patients with the latter condition typically display high plasma/serum Cn concentrations [114]. Although this finding may be incidental, there remains the possibility of a genetic association between these two conditions.

In order to further explore the possible contributions of age differences between the GM1T2 and HC groups studied, we accessed an already available very extensive ^1^H NMR-based dataset which reported blood serum concentrations of most of the metabolites determined in the current study, for both an 11–12-year-old childhood cohort and their parental ‘controls’. From this dataset, child:adult fold-changes for Ile-normalized mean levels available serum biomolecules were calculated, and these were then compared with corresponding Ile-normalized metabolite GM1T2:HC fold-changes and their 95% CIs computed from our 700 MHz ^1^H NMR-based dataset. These comparisons confirmed statistically significant GM1T2 disease-mediated upregulations in the BCAAs leucine and valine, the aromatic amino acids tyrosine, phenylalanine and histidine, and Cn, lactate and HDL-associated phospholipids (the marked increases in Cn and lactate concentrations being very highly significant). Furthermore, some evidence for a significant downregulation in plasma citrate was also obtained.

Similarly, the marked upregulations found for a wide range of plasma amino acids in GM1T2 patients (with the exception of alanine and Ile), may also arise from an increased level of dietary protein sources in this unfasted participant cohort. Indeed, the feeding of a low-protein diet to rats was found to decrease plasma levels of essential amino acids during an absorptive state, and this observation provided evidence that diminished plasma amino acid levels may serve as an early signal of protein deficiency [115]. However, as with the above considerations for plasma creatinine, in the current study the potentially complicating influence of diet appears to be highly unlikely in view of the substantial corresponding downregulations observed for total lipoprotein-associated TAGs discovered in this group (fold-change 0.75 for CS-normalized data).

It should also be noted that this study was limited by the small size of the GM1T2 patient cohort sample donors, and this arises from the very rare nature of this disease, and hence the extremely limited availability of such sample donors. Although such small sample sizes are a frequent concern in metabolomics investigations of LSDs, and for this study prior power calculations performed recommended a minimum sample size of approximately 30 participants per group [21], fortunately the application of both UV and MV metabolomics analysis options still found very highly statistically significant metabolic distinctions between this classification and the HC one. These observations arose from the highly profound biomolecular differences observed between their plasma ^1^H NMR profiles. Notwithstanding, the reliabilities and accuracies of the results acquired may be impacted by this GM1T2 sample size limitation, and therefore should be interpreted with some caution.

Further evidentiary support was provided by the data analysis strategies applied, which were highly rigorous, and included the primary tracking of sample outliers with PCA. Furthermore, robust Welch tests were implemented for the univariate ANOVA models employed, and Bonferroni- corrections were then applied for the performance of post-hoc tests in order to avoid potential issues with false positives (type I errors).

Another limitation of the study is that GM1T2 patients commonly receive dietary supplements, and these may engender some phenotypically-unrelated differences between their plasma ^1^H NMR profiles and those of the HC group, as indeed may the possible differential dietary habits of these two participant groups (the authors were informed that one of the GM1T2 participants was receiving vitamin B6).

An additional complication is potential interferences mediated by the therapeutic agents received by the GM1T2 participant cohort; however, it should be noted that many of these patients continuously and commonly receive a range of such medications, including miglustat (substrate inhibitor usually for the treatment of late-onset GM1 gangliosidosis), anticonvulsants such as valproate and clonidine for a range of GM1 comorbidities, and tolperisone as a muscle relaxant, etc. In principle, the drug therapies administered to the GM1T2 cohort may have the ability to exert significant influences on the plasma levels of endogenous metabolites; however, extensive studies addressing the effect of these drugs on the human plasma and urinary metabolomes, most especially those of LSD patients, remain unperformed. Although one of the GM1T2 participants was not undergoing any therapy, the remainder were receiving a combination of medications (documented in Section 2.1). From available literature data, the maximal observed blood plasma concentrations of these drugs are provided in Section S1 of the Supplementary Materials. From these data, potential ^1^H NMR-detectable interferences arising from the resonances of these xenobiotics and/or their metabolites in the GM1T2 plasma profiles are only likely to be ascribable to valproate or levetiracetam, since maximal plasma levels of the majority of other therapeutic agents featured are below or close to the limit of ^1^H NMR detection for this technique, even at an operating frequency of 700 MHz. However, one clear observation was the complete absence of resonances attributable to drugs and/or metabolites in the ^1^H NMR profiles of all GM1T2 blood plasma samples acquired. This was confirmed via the acquisition of 2D COSY and TOCSY spectra of the samples concerned in view of the possible or likely overlap of these signals with those of selected endogenous biomolecules. The urinary excretion of both miglustat and valproate, along with metabolites of the latter, have been previously explored in the urinary ^1^H NMR profiles of patients with NPC1 disease [20]; for this study, a similar urinalysis confirmed that low levels of miglustat and valproate/valproate glucuronide were present in the urine of the single GM1T2 patients receiving these therapies. These data will be presented elsewhere.

Notwithstanding, as required, none of the HC participants were taking any medications or supplements (on the participant information sheet for this study, they were instructed not to for a minimum period of 7 days prior to biofluid sample collections). As expected, signals ascribable to or derived from xenobiotics, e.g., common analgesics such as aspirin, paracetamol, and ibuprofen, were also not found in any of the HC group participant spectra obtained, and nor were those of any metabolites of these drugs. Similarly, ethanol was undetectable in these samples.

Confirmation of the importance and discriminatory potential of the biomarkers found in this study will be best achieved by the multicomponent ^1^H NMR analysis of additional samples collected from very rare GM1T2 patients, and also perhaps an improved participant age range matching for the HC participants.

Finally, an additional potential limitation is complications with the employment of enrichment ratios for the computation of pathway activities using QMSEA. Indeed, this approach does not consider the uncertainties or errors associated with assigning metabolites to specified pathways. As a hypothetical example, the ascription of 3 ^1^H NMR-determined metabolites to a single pathway containing a total of 8 metabolites will yield a relatively high enrichment ratio of 3/8; however, if all three of these pathway-linked metabolites are also active in alternative pathways, then confidence in the implication of this pathway from metabolomics datasets will be limited. This limitation is reviewed and discussed in [116] in detail.

## 6. Conclusions

This study provides valuable information regarding the abnormal metabolic status of GM1T2 blood plasma, and the probable dysfunctional metabolic pathways giving rise to them. Indeed, significant GM1T2-mediated upregulations in a series of free amino acids (notably BCAAs, the neurotransmitter glutamate and its glutamine precursor, and aromatic classes), Cn, lactate, citrate, and further metabolites, along with downregulations in total lipoprotein-associated TAGs and alanine, were found. Application of a range of MV analysis techniques and CITs all demonstrated clear segregations between the GM1T2 and HC metabolic profiles for both full ^1^H NMR ISB and assigned metabolite variable datasets. QMSEA supported important roles for the involvement of propanoate metabolism, BCAA and ethanol degradation, FA biosynthesis, and other pathways involved in the pathogenesis of GM1T2 disease. Furthermore, this strategy was employed to explore potential organ, tissue, cellular, and sub-cellular locations of pathological activities associated with these altered metabolite sets, and found that fibroblasts > Golgi apparatus > mitochondria > spleen ≈ skeletal muscle ≈ muscle in general were the major contributors to GM1T2 disease pathology in that order of importance. Indeed, these represent key sites for this condition’s clinical features and symptomatic developments. Overall, the results acquired provided evidence for highly significant alterations to energy demand and metabolic pathways, both of which are major features of the mechanisms of GM1T2 disorder pathogenesis. Indeed, combinations of a series of potential biomarkers in the form of a disturbed metabolic ‘signature’ or pattern offers major advantages over the use of only a single, targeted biomarker for diagnostic or prognostic evaluation purposes in LSDs. Therefore, global metabolomics profiling of biofluid samples may provide a valuable innovative probe for improving our understanding of these mechanisms, and also for the seeking and validation of potential diagnostic biomarkers for this debilitating condition. Such investigations pave the way for future explorations of metabolic imbalances found in GM1 gangliosidosis and other LSDs, primarily those focused on (1) the discovery of longitudinal responses (i.e., repeated biofluid samplings of each patient over time) in order to reliably and rigorously monitor disease severity and progression, and (2) future successful validation of the biomarkers of disease activities and progression found here in blood plasma, and perhaps also further biofluids such as CSF and/or urine, both in animal models and ultimately humans. Such validation processes in humans may now indeed be realized in view of the advent of the availability of a potentially successful GT treatment for human GM1 disease.

Finally, this study confirms the value of multicomponent ^1^H NMR spectroscopic analysis as a rapid and virtually non-invasive strategy to seek and detect biomarkers for GM1T2 disease, information which may be transferable to the diagnosis and prognostic monitoring of other LSDs. Indeed, this knowledge may facilitate the design, development, and operation of new pattern recognition-based chemopathological tracking methods for the diagnosis and such disorders, and also provide state-of-the-art information regarding dysfunctional metabolic pathways, and the sources and localizations of those involved.

## Figures and Tables

**Table 1:** Chemical shift values, multiplicities, and assignments for resonances present in the ^1^H NMR profiles of blood plasma collected from GM1T2 and HC participants (Figure 1).

Resonance Code	Chemical Shift (δ/ppm)	Multiplicity	Assignment	Median STN Value *
1	0.92	*broad*	Very-low-density-lipoprotein (vLDL)/low-density-lipoprotein (LDL) TAG-terminal-CH_3_ functions	413
2	0.926 and 0.997	*t* and *d*	Isoleucine-CH_3_	82 and 118
2	0.949	*t*	Leucine-CH_3_	108
2	0.98 and 1.03	2 × *d*	Valine-CH_3′_s	226 and 253
3	1.10–1.30	*broad*	vLDL/LDL-bulk-chain-(-CH_2_-)n	599
4	1.32	*d*	Lactate-CH_3_	501
5	1.48	*d*	Alanine-CH_3_	382
6	1.55	*broad*	Lipoprotein TAG-CH_2_CH_2_CO	157
7	1.74	*broad m*	Arginine-γ-CH_2_/Lysine-δ-CH_2_ protein residues	94
8	1.92	*s*	Acetate-CH_3_	198
9	2.03	*broad*	Acute-phase glycoprotein-carbohydrate side-chain N-acetyl-sugar-CH_3_ I (APG-I)/TAG-CH_2_-CH = CH-	281
9	2.07	*broad*	Acute-phase glycoprotein-carbohydrate side-chain N-acetyl-sugar-CH_3_ II (APG-II)/TAG-CH_2_-CH = CH-	386
10	2.12	*m*	Glutamine-β-CH_2_	122
11	2.21	*m*	Lipoprotein TAG-CH_2_-CO_2_^-^/Acetone-CH_3_	111
12	2.43	*m*	Glutamine-γ-CH_2_	169
13	2.53	½ *dd* (AB coupling pattern)	Citrate-CH_2A/B_	107
14	2.75	*d*	Lipoprotein TAG-CH = CH-CH_2_-CH = CH/Citrate-CH_2A/B_	89
15	2.97	*broad*	Albumin lysine residue-ε-CH_2_	82
16	3.02	*t*/*s*	Free lysine-ε-CH_2_/Creatine- and Creatinine-N-CH_3_	151
17	3.19	*broad*	High-density-lipoprotein phospholipid choline head-group-N(CH)_3_^+^	459
18	3.22	*m*	Glucose-C2H	478
19	3.39	*m*	Taurine-CH_2_SO_3_^2-^	279
20	3.41	*m*	Glucose-C2-6H	463
21	3.43	*m*	Glucose-β-C2/5-α-C3/5-H	473
22	3.57	*m*	Glucose-β-C2/5-α-C3/5-H	539
23	3.62	*m*	Unassigned	133
24	3.71	*m*	Glucose-α-C3/6- β-C6-H	434
25	3.78	*m*	Glucose-α-C3/6- β-C6-H	451
26	3.82	*m*	Glucose-α-C4/6- β-C4/6-H	444
27	3.89	*m*	Glucose-α-C4/6- β-C4/6-H	421
Cr/PCr	3.94	*s*	Creatine/Phosphocreatine-CH_2_	151
28	3.99	*ms*	Tyrosine-/Histidine-/Phenylalanine-α-CH’s	80
29	4.05	*s*	Creatinine-CH_2_	133
30	4.14	*q*	Lactate-CH	135
31	4.63	*d*	β-Glucose-CH	594
32	4.80	*s*	Residual H_2_O/HO^2^H	n/a
33	5.25	*d*	α-Glucose-CH	341
34	5.27	*broad*	Lipoprotein TAG-CH = CH-	555
35	5.80	*broad*	Urea-CO-NH_2_	49
36	6.89	*m*	Tyrosine-CH	57
37	7.01	*m*	Phenylalanine-CH	34
38	7.08	*s*	Histidine-CH	83
39	7.19	*m*	Tyrosine-CH	63
40	7.30	*m*	Phenylalanine-CH	50
41	7.41	*m*	Phenylalanine-CH	30
42	7.80	*s*	Histidine-CH	79
43	8.07	*broad*	Protein aromatic amino acid residue(s)	45
44	8.45	*s*	Formate-CH	63

Abbreviations: s, d, dd, t, q, and m, singlet, doublet, doublet of doublets, triplet, quartet, and multiplet, respectively. Median 1H NMR signal-to-noise (STN) ratios were determined by the method described in Section 2.5. * The lower limit of quantification (LLOQ) value was defined as 10 times the 1H NMR noise value.

**Table 2:** Statistical significance and nature of differences between the ^1^H NMR-detectable plasma metabolite levels (as ^1^H NMR resonance intensity equivalents) of GM1T2 and HC participants.

Metabolite	Regulation Status (↑/↓) *	Fold-Change **	Age-Related Fold-Change (HC data) ***	WT *p* Value	Bonferroni-Corrected WT *p*-Value
Total TAGs	↓	0.75	0.808 (total TAGs)0.842 (total FAs)	4.50 × 10^−5^	1.12 × 10^−3^
Isoleucine	↑	1.04	0.947	ns	ns
Leucine	↑	1.37	0.935	4.67 × 10^−4^	1.17 × 10^−2^
Valine	↑	2.10	0.958	<10^−6^	<2.50 × 10^−5^
3-AIB	↑	1.21	na	9.02 × 10^−3^	ns
Alanine	↓	0.87	0.973	4.37 × 10^−2^	ns
Acetate	↓	0.96	0.882	ns	ns
APG-I/TAGs	↓	0.91	0.930	ns	ns
APG-II/TAGs	↓	0.93	na	3.41 × 10^−2^	ns
Glutamine	↑	1.20	1.052	1.16 × 10^−4^	2.90 × 10^−3^
Glutamate	↑	1.14	1.308****	4.70 × 10^−4^	1.17 × 10^−2^
Citrate	↑	1.16	1.153	1.16 × 10^−3^	2.90 × 10^−2^
HDL-PLs	↑	1.03	0.859 (total PGs)/0.870 (total cholines)	ns	ns
α-/β-Glucose	↑	1.14	0.998	2.91 × 10^−2^	ns
Taurine	↑	1.13	1.073 ****	3.58 × 10^−2^	ns
Cr/PCr	↑	1.48	0.998 (Cr only) ****	2.80 × 10^−3^	ns
Cn	↑	2.65	0.727	2.18 × 10^−5^	5.45 × 10^−4^
Lactate	↑	4.19	1.103	3.27 × 10^−6^	8.17 × 10^−5^
Threonine	↑	1.16	1.225 ****	ns	ns
Urea	↑	1.66	0.473 ****	1.55 × 10^−3^	3.87 × 10^−2^
Tyrosine	↑	1.62	1.000	7.41 × 10^−5^	1.85 × 10^−3^
Phenylalanine	↑	1.52	0.918	1.26 × 10^−3^	3.15 × 10^−2^
Histidine	↑	1.91	1.000	2.57 × 10^−4^	6.42 × 10^−3^
PAAR	↑	1.96	na	2.78 × 10^−4^	6.95 × 10^−3^
Formate	↑	1.54	na	4.05 × 10^−3^	ns

Both uncorrected and Bonferroni-corrected ANOVA Welch test (WT) significance (*p*) values are provided. Fold-changes were calculated as the ratio of the GM1T2 group mean value to that of the HC one for each metabolite predictor considered. In addition, provided are age-related healthy human blood serum metabolite fold-change data calculated from Ref. [23] to permit evaluations of the magnitude of metabolite concentration differences arising from contrasting age groups alone. Abbreviations: * ↑ and ↓ indicate up- and downregulations of metabolite concentrations, respectively, in GM1T2 disease; ns, not statistically significant; APG-I and II, magnetically-distinguishable -NHCOCH_3_ environments of *N*-acetylneuraminate and *N*-acetylglucosamine residues present in the molecularly-mobile carbohydrate side-chains of acute-phase glycoproteins (their corresponding ISB intensities are those of broad resonances centered at δ = 2.03 and 2.07 ppm, respectively, albeit also with some interferences arising from TAG-CH_2_-CH=CH- signal(s), more so for the former); FAs, fatty acids; HDL-PLs, high-density-lipoprotein phospholipids (choline head-group -N(CH_3_)_3_^+^ resonance); PAAR, broad protein aromatic amino acid resonance (δ = 8.03–8.10 ppm); PGs, phosphoglycerides; na, not available. ** Indicates fold-change for CS-normalized datasets. *** Indicates absolute serum fold-changes for two healthy human age groups, i.e., child:adult values [23]. **** Unavailable in Ref. [23], and therefore calculated from data available in Ref. [22].

**Table 3:** Statistical significance and nature of differences between the ^1^H NMR-detectable, Ile-normalized (Ile-N) plasma metabolite levels of GM1T2 and HC participants.

Metabolite	Ile-N Fold-Change ± 95% CIs	Age-Related Ile-N Fold-Change (HC data)	Ile-N WT *p* Value	Ile-N Bonferroni-Corrected WT *p*-Value
Total TAGs	0.74 ± 0.22	0.85	5.53 × 10^−3^	ns
Isoleucine	na	na	n/a	n/a
Leucine	1.32 ± 0.04	0.99	2.21 × 10^−5^	5.30 × 10^−4^
Valine	2.00 ± 0.05	1.01	<10^−6^	<2.5 × 10^−4^
3-AIB	1.18 ± 0.16	na	ns	ns
Alanine	0.86 ± 0.22	1.03	0.079	ns
Acetate	0.92 ± 0.16	0.96	ns	ns
APG-I/TAGs	0.88 ± 0.27	0.98	4.62 × 10^−2^	ns
APG-II/TAGs	0.89 ± 0.11	na	3.53 × 10^−2^	ns
Glutamine	1.16 ± 0.09	1.11	6.83 × 10^−3^	ns
Glutamate	1.11 ± 0.39	na	ns	ns
Citrate	1.12 ± 0.09	1.22	0.057	ns
HDL-PLs	1.01 ± 0.19	0.89 (total PGs)/0.92 (total cholines)	ns	ns
α-/β-Glucose	1.06 ± 0.10	1.05	ns	ns
Taurine	1.08 ± 0.11	na	ns	ns
Cr/PCr	2.59 ± 0.10	na	<10^−6^	<2.50 × 10^−4^
Cn	2.50 ± 0.11	0.77	3.67 × 10^−5^	8.81 × 10^−4^
Lactate	3.98 ± 0.40	1.16	4.98 × 10^−6^	1.19 × 10^−4^
Threonine	1.11 ± 0.33	na	ns	ns
Urea	1.54 ± 0.21	na	2.37 × 10^−3^	0.057
Tyrosine	1.53 ± 0.14	1.06	2.00 × 10^−4^	9.60 x 10^−3^
Phenylalanine	1.43 ± 0.19	0.97	2.45 × 10^−3^	0.059
Histidine	1.78 ± 0.19	1.06	4.06 × 10^−4^	9.74 × 10^−3^
PAAR	2.11 ± 0.17	na	2.10 × 10^−4^	5.04 × 10^−3^

Both uncorrected and Bonferroni-corrected ANOVA Welch test (WT) significance (*p*) values are provided, as in Table 2. In addition, listed are fold-changes computed from the GM1T2:HC ratio of mean Ile-N metabolite levels, along with their estimated 95% CIs. Corresponding Ile-N, age-related child:adult fold-change data calculated from the extensive Ref. [23] dataset for specified metabolites are also provided. Abbreviations: as Table 1 and Table 2.

**Table 4:** Loadings vectors of metabolites showing their significant contributions towards PCs 1–5 for the Model 2 dataset (corresponding squared cosine values in brackets). Biomolecular predictors with loadings vectors of magnitudes within the ±0.40 range were not considered (nc), since they are only included as significant contributors if they lie outside it [17].

PC	PC1	PC2	PC3	PC4	PC5
Eigenvalue	9.69	3.61	2.91	2.40	1.05
Total TAGs	−0.50 (0.25)	−0.79 (0.62)	nc	nc	nc
Isoleucine	nc	0.70 (0.49)	nc	nc	nc
Leucine	nc	0.94 (0.88)	nc	nc	nc
Valine	0.47 (0.22)	0.72 (0.53)	nc	nc	nc
3-AIB	0.53 (0.28)	nc	nc	nc	0.69 (0.47)
Alanine	nc	nc	nc	0.40 (0.16)	−0.62 (0.38)
Acetate	0.45 (0.20)	nc	0.48 (0.23)	nc	0.52 (0.28)
APG-1	nc	nc	nc	0.81 (0.65)	nc
APG-2	nc	nc	nc	0.87 (0.76)	nc
Glutamine	nc	0.88 (0.78)	nc	nc	nc
Glutamate	0.53 (0.28)	nc	0.60 (0.36)	nc	nc
Citrate	nc	0.42 (0.18)	0.60 (0.36)	nc	0.45 (0.20)
HDL-PLs	−0.42 (0.17)	nc	nc	−0.83 (0.69)	nc
Taurine	nc	nc	0.74 (0.55)	nc	nc
Cr/PCr	0.65 (0.43)	nc	0.60 (0.36)	nc	nc
Cn	0.50 (0.25)	nc	0.75 (0.57)	nc	nc
Lactate	0.47 (0.22)	nc	0.75 (0.57)	nc	nc
Threonine	nc	nc	0.87 (0.76)	nc	nc
Glucose	nc	0.56 (0.32)	−0.69 (0.47)	nc	nc
Urea	0.92 (0.85)	nc	nc	nc	nc
Tyrosine	0.93 (0.87)	nc	nc	nc	nc
Phenylalanine	0.95 (0.90)	nc	nc	nc	nc
Histidine	0.93 (0.87)	nc	nc	nc	nc
PAAR	0.92 (0.84)	nc	nc	nc	nc
Formate	0.93 (0.86)	nc	nc	nc	nc

**Table 5:** Mean±SEM predictive accuracies for the training, test and total sets in a PLS-DA-based validation model involving a ‘hold-out’ sample of *n* = 9 plasma samples. The mean validation set classification success score is also provided.

	GM1T2 Disease	HC	Total
Training Set (*n* = 19)	89.35 ± 1.92%	99.91 ± 0.09%	97.95 ± 0.31%
Test Set (*n* = 9)	83.22 ± 3.17%	99.06 ± 0.53%	94.66 ± 0.95%
Mean Validation Set Classification Success Score (*n* = 9)	8.75/9.00 (97.22%)		

**Table 6:** Enrichment overview of QMSEA of the key biomarker dataset, showing pathway ‘hits’, computed Q statistic parameters, and their corresponding *p* values. All calculated *p* values indicated were corrected for multiple comparisons via the Holm–Bonferroni false discovery rate (FDR) strategy.

Pathway	Total Metabolites	Hits	Q Statistic	FDR-Corrected *p*-value
Propanoate Metabolism	42	2	59.66	7.12 × 10^−12^
Valine, Leucine and Isoleucine Degradation	60	4	37.97	1.33 × 10^−9^
Ethanol Degradation	19	1	53.21	3.79 × 10^−6^
Fatty Acid Biosynthesis	35	1	53.21	3.79 × 10^−6^
Amino-sugar Metabolism	33	3	27.23	1.90 × 10^−5^
Aspartate Metabolism	35	3	27.23	1.90 × 10^−5^
Seleno-Amino Acid Metabolism	28	1	45.64	3.21 × 10^−5^
Glutathione Metabolism	21	2	36.41	4.25 × 10^−5^
Alanine Metabolism	17	2	36.41	4.25 × 10^−5^
Glucose-Alanine Cycle	13	3	27.72	0.00017
Glutamate Metabolism	49	3	27.79	0.00030
Pyruvate Metabolism	48	2	22.98	0.008
Warburg Effect	58	5	19.02	0.014
Gluconeogenesis	35	2	20.28	0.016
Cysteine Metabolism	26	1	16.29	0.037
Lysine Degradation	30	1	16.29	0.037
Arachidonic Acid Metabolism	69	1	16.29	0.037
Malate-Aspartate Shuttle	10	1	16.29	0.037
Urea Cycle	29	4	13.69	0.037
Transfer of Acetyl Groups into Mitochondria	22	2	10.95	0.049

**Table 7:** Overview of organ, tissue, cellular, and sub-cellular localization-based metabolite sets onset by GM1T2-mediated pathological dysfunctions via QMSEA with fold enrichments and corresponding *p* values (the library available contained 73 entries). Up- or downregulated plasma metabolite concentrations contributing towards each localization are indicated. All calculated *p* values indicated were corrected for multiple comparisons via the Holm–Bonferroni strategy.

Site	Total Metabolites Involved	Number of Hits	FDR-Adjusted *p* Value	Metabolites Featured (Up-/Down Regulation in GM1T2 Disease Observed)
Fibroblasts	183	1	1.31 × 10^−12^	Valine↑
Golgi Apparatus	14	1	3.04 × 10^−6^	Acetate↓
Mitochondria	98	5	4.74 × 10^−3^	Valine↑/Acetate↓/Urea↑/ Glutamine↑/Leucine↑
Spleen	170	3	3.33 × 10^−2^	Glutamate↑/Creatinine↑/ Glutamine↑
Skeletal Muscle	123	5	3.33 × 10^−2^	Glutamate↑/Creatinine↑/ Taurine↑/Glutamine↑/Leucine↑
Muscle	160	6	3.33 × 10^−2^	Glutamate↑/Creatinine↑/ Glucose↑/Taurine↑/Leucine↑

## Data Availability

Data supporting reported results can be obtained from the correspondence author (mgrootveld@dmu.ac.uk).

## Supplementary Materials

The following are available online at https://www.mdpi.com/2073-4409/10/3/572/s1, Table S1: Tabular representation of dysfunctional PMS enzymes, and metabolite transport and transfer processes indicated for involvement in GM1T2 pathology. Clearly, BCAA catabolism represents a major feature of the pathogenesis of this disease, Section S1: Scientific Literature Data Available on the Maximal Blood Plasma Concentrations of Drugs Received by the GM1T2 Patient Cohort.
