# Supplementary material for: Rapid Identification of New Biomarkers for the Classification of GM1 Type 2 Gangliosidosis Using an Unbiased 1H NMR-Linked Metabolomics Strategy
Source: Cells. 2021 Mar 5;10(3):572. doi: 10.3390/cells10030572 (PMC7998791; doi:10.3390/cells10030572)
Supplement: Supplementary file 1 [file cells-10-00572-s001.pdf]

Rapid Identification of New Biomarkers for the Classification of GM1 Type 2 Gangliosidosis using an Unbiased ^{1}H NMR-Linked Metabolomics Strategy

Benita C. Percival and Martin Grootveld*

Leicester School of Pharmacy, De Montfort University, The Gateway, Leicester LE1 9BH, UK

SUPPLEMENTARY MATERIALS

***Correspondence: mgrootveld@dmu.ac.uk; Tel.: +44-(0)116-250-6443**

Section S1: Scientific Literature Data Available on the Maximal Blood Plasma Concentrations of Drugs Received by the GM1T2 Patient Cohort

These values are: miglustat, *ca.* 10 μmol./L [S1]; valproate, *ca.* 1.0 mmol./L [S2]; clonidine, sub-μmol./L [S3]; lamotrigine, *ca.* 5 μmol./L [S4]; tolperisone, *ca.* 0.5 μmol./L [S5]; levetiracetam, *ca.* 300-400 μmol./L [S6]; albuterol sulphate, sub-μmol./L [S7]; lamotrigine, *ca.* 5 μmol./L [S8]; cyclopentolate, sub-μmol./L [S9]; and diazepam, approaching 10 μmol./L [S10].

Supplementary Materials References

[S1] Spieker, E.; Wagner-Redeker, W.; Dingemanse, J. Validated LC–MS/MS method for the quantitative determination of the glucosylceramide synthase inhibitor miglustat in mouse plasma and human plasma and its application to a pharmacokinetic study. *J. Pharm. Biomed. Anal.* **2012**, *59*, 123-129.

[S2] Turnbull, D. M.; Rawlins, M. D.; Weightman, D.; Chadwick, D. W. Plasma concentrations of sodium valproate: Their clinical value. *Ann. Neurol.* **1983**, *14*, 38-42. https://doi.org/10.1002/ana.410140107

[S3] Davies, D. S. Wing, L. M. H.; Reid, J. L.; Neill, E.; Tippett, P.; Dollery, C. T. Pharmacokinetics and concentration-effect relationships of intravenous and oral clonidine. *Clin. Pharmacol. Therapeut.* **1977**, *21*, doi: 10.1002/cpt1977215593.

[S4] Suzuki, T.; Mihara, K.; Nagai, G.; Kagawa, S.; Nakamura, A.; Nemoto, K.; Kondo, T. A high plasma lamotrigine concentration at week 2 as a risk factor for lamotrigine-related rash. *Therapeut. Drug Monitor.* **2020**, *42*(4), 631-635. doi: 10.1097/FTD.0000000000000733

[S5] Choi, C-I.; Park, J-I.; Lee, H-I.; Lee, Y-J.; Jang, C-G.; Bae, J-W.; Lee, S-Y. Determination of tolperisone in human plasma by liquid chromatography/tandem mass spectrometry for clinical application. *J. Chromatog. B.* **2012**, *911*, 59-63. ISSN 1570-0232, https://doi.org/10.1016/j.jchromb.2012.10.027..

[S6] Patsalos, P. N.; Berry, D. J.; Bourgeois, B. F.; Cloyd, J. C.; Glauser, T. A.; Johannessen, S. I.; Leppik, I. E.; Tomson, T,; Perucca, E. Antiepileptic drugs-best practice guidelines for therapeutic drug monitoring: a position paper by the subcommission on therapeutic drug monitoring. *ILAE Commission on Therapeut. Strat. Epilepsia* **2008**, *49*(7), 1239-1276.

[S7] Mohamed, M. H. N.; Lima, J. J.; Eberle, L. V.; Self, T. H.; Johnson, J. A. Effects of gender and race on albuterol pharmacokinetics. *Pharmacotherapy: J. Human Pharmacol. Drug Therap.* **1999**, *19*, 157-161. https://doi.org/10.1592/phco.19.3.157.30925

[S8] Lahdes, K.; Huupponen, R.; Kaila, T.; Monti, D.; Saettone, M.; Salminen, L. Plasma concentrations and ocular effects of cyclopentolate after ocular application of three formulations. *Br. J. Clin. Pharmacol.* **1993**, *35*, 479-483. https://doi.org/10.1111/j.1365-2125.1993.tb04173.x

[S9] Bond, A. J.; Hailey, D. M.; Lader, M. H. Plasma concentrations of benzodiazepines. *Br. J. Clin. Pharmacol.* **1977**, 4(1), 51-56. doi: 10.1111/j.1365-2125.1977.tb00666.x. PMID: 14659; PMCID: PMC1428979.

[S10] Tanimoto, S.; Pesco Koplowitz, L.; Lowenthal, R. E.; Koplowitz, B.; Rabinowicz, A. L.; Carrazana, E. Evaluation of pharmacokinetics and dose Proportionality of diazepam after intranasal administration of NRL-1 to healthy volunteers. *Clin. Pharmacol. Drug Develop.* **2020**, *9*, 719-727. https://doi.org/10.1002/cpdd.767

Supplementary Materials Table

Table S1. Tabular representation of dysfunctional PMS enzymes, and metabolite transport and transfer processes indicated for involvement in GM1T2 pathology. Clearly, BCAA catabolism represents a major feature of the pathogenesis of this disease.

**Enzyme/Process**	**Total Compounds**	**Hits**	**Q Statistic**	**FDR *p* value**	**Pathway**
2-Oxoisovalerate dehydrogenase (acylating: 3-methyl-2-oxobutanoate), mitochondrial	2	1	80.32	7.35×10^{-13}	BCAA degradation
3-Amino-isobutyrate transport	2	1	80.32	7.35×10^{-13}	BCAA degradation
3-Amino-isobutyrate transport, mitochondrial	2	1	80.32	7.35×10^{-13}	BCAA degradation
3-Hydroxyacyl-CoA dehydratase (3-hydroxyisobutyryl-CoA) (mitochondria)	2	1	80.32	7.35×10^{-13}	Butanoate metabolism
3-Hydroxyisobutyrate dehydrogenase, mitochondrial	2	1	80.32	7.35×10^{-13}	BCAA degradation
3-Hydroxyisobutyryl-CoA hydrolase, mitochondrial	2	1	80.32	7.35×10^{-13}	BCAA degradation/β-Alanine metabolism/Propanoate metabolism
Acyl-CoA dehydrogenase (isobutyryl-CoA), mitochondrial	2	1	80.32	7.35×10^{-13}	First stage of FA metabolism
L-3-Amino-isobutanoate exchange	2	1	80.32	7.35×10^{-13}	BCAA degradation
L-3-Aminoisobutyrate transaminase, mitochondrial	2	1	80.32	7.35×10^{-13}	BCAA degradation
Malonate-semialdehyde dehydrogenase (acetylating), mitochondrial	2	1	80.32	7.35×10^{-13}	Inositol metabolism/Alanine and Aspartate metabolism/β-Alanine metabolism/Propanoate metabolism.
Methylmalonate-semialdehyde dehydrogenase	2	1	80.32	7.35×10^{-13}	Inositol metabolism/BCAA degradation/Propanoate metabolism.
Methylmalonyl-CoA mutase	8	1	25.86	0.012	Degradation of odd-chain FAs, valine, isoleucine, methionine, threonine and cholesterol.

					Funneling of metabolites arising from these amino acids into the TCA cycle.
Propionyl-CoA carboxylase, mitochondrial	8	1	25.86	0.012	Propionyl CoA (PCoA) is the end- product of metabolism of odd-chain FAs, and a methyl-branched FA metabolite.<br><br>PCoA is a major valine metabolite, and along with acetyl-CoA, is also a metabolite of isoleucine and methionine.
